# Relationship Between COVID-19 Infection and Risk Perception, Knowledge, Attitude, and Four Nonpharmaceutical Interventions During the Late Period of the COVID-19 Epidemic in China: Online Cross-Sectional Survey of 8158 Adults

**DOI:** 10.2196/21372

**Published:** 2020-11-13

**Authors:** Hong Xu, Yong Gan, Daikun Zheng, Bo Wu, Xian Zhu, Chang Xu, Chenglu Liu, Zhou Tao, Yaoyue Hu, Min Chen, Mingjing Li, Zuxun Lu, Jack Chen

**Affiliations:** 1 School of Public Health Tongji Medical College Huazhong University of Science and Technology Wuhai China; 2 School of Public Health and Management Chongqing Medical University Chongqing China; 3 Chongqing Three Gorges Medical College Chongqing China; 4 Wanzhou District Center for Disease Control and Prevention Chongqing China; 5 Ingham Institute & University of New South Wales Sydney Australia

**Keywords:** COVID-19, nonpharmaceutical personal interventions, NPI, public health, mask wearing, intervention, infection, risk perception, knowledge, attitude, online survey, China

## Abstract

**Background:**

So far, there have been no published population studies on the relationship between a COVID-19 infection and public risk perception, information source, knowledge, attitude, and behaviors during the COVID-19 outbreak in China.

**Objective:**

This study aims to understand the relationships between COVID-19 infection; four personal nonpharmaceutical interventions (NPIs; handwashing, proper coughing habits, social distancing, and mask wearing); and public risk perception, knowledge, attitude, and other social demographic variables.

**Methods:**

An online survey of 8158 Chinese adults between February 22 and March 5, 2020, was conducted. Bivariate associations between categorical variables were examined using Fisher exact test. We also explored the determinants of four NPIs as well as their association with COVID-19 infection using logistic regression.

**Results:**

Of 8158 adults included, 57 (0.73%) were infected with COVID-19. The overwhelming majority of respondents showed a positive attitude (n=8094, 99.2%), positive risk perception (n=8146, 99.9%), and high knowledge levels that were among the strongest predictors of the four adopted NPIs (handwashing: n=7895, 96.8%; proper coughing: 5997/6444, 93.1%; social distancing: n=7104/8158, 87.1%; and mask wearing: 5011/5120, 97.9%). There was an increased risk of COVID-19 infection for those who did not wash their hands (2.28% vs 0.65%; risk ratio [RR] 3.53, 95% CI 1.53-8.15; *P*=.009), did not practice proper coughing (1.79% vs 0.73%; RR 2.44, 95% CI 1.15-5.15; *P*=.03), did not practice social distancing (1.52% vs 0.58%; RR 2.63, 95% CI 1.48-4.67; *P*=.002), and did not wear a mask (7.41% vs 0.6%; RR 12.38, 95% CI 5.81-26.36; *P*<.001). For those who did practice all other three NPIs, wearing a mask was associated with a significantly reduced risk of infection compared to those who did not wear a mask (0.6% vs 16.7%; *P*=.04). Similarly, for those who did not practice all or part of the other three NPIs, wearing a mask was also associated with a significantly reduced risk of infection. In a penalized logistic regression model including all four NPIs, wearing a mask was the only significant predictor of COVID-19 infection among the four NPIs (odds ratio 7.20, 95% CI 2.24-23.11; *P*<.001).

**Conclusions:**

We found high levels of risk perception, positive attitude, desirable knowledge, as well as a high level of adopting the four NPIs. The relevant knowledge, risk perception, and attitude were strong predictors of adapting the four NPIs. Mask wearing, among the four personal NPIs, was the most effective protective measure against COVID-19 infection, with added preventive effect among those who practiced all or part of the other three NPIs.

## Introduction

The unprecedented COVID-19 global pandemic [1] has changed the way our society operates. The total confirmed cases and deaths worldwide increased at an alarming rate [2]. The availability of an effective vaccine may still be many months away [3,4], and there is no consensus on the use of antiviral drugs and other therapeutic agents [5,6]. Meanwhile, the best hope for reducing mortality is societal preventative measures and providing timely and optimal critical care. As the list of countries in the grip of the rapid spread of COVID-19 is growing, many countries are, or will be, at the brink of further overwhelmed health care systems. Many countries have strengthened their nonpharmaceutical interventions (NPIs) to flatten the curve and reduce casualties [7]. For the NPIs to be effective, one of the critical conditions is the public’s active participation and compliance. Since the lockdown of Wuhan City on January 23, 2019, China was the first country to introduce NPIs with strict measures such as the lockdown of cities and counties; compulsory mask wearing; isolation of suspicious cases; screening and contact tracing; quarantining people from high risk areas for 14 days; as well as promoting handwashing, proper coughing habits, social distancing, and self-isolation. However, there was no published evidence on relationships between a COVID-19 infection, the Chinese public risk perception, information source, knowledge, attitude, and personal NPIs during the middle to the end of the epidemic.

Between February 22, 2020, to March 5, 2020 (the late period of the COVID-19 epidemic in China) [2], we conducted an online cross-sectional survey of Chinese residents to understand risk perceptions, information source, knowledge, attitude, and practice of the Chinese public after the COVID-19 outbreak; explore the determinants associated with the key personal NPIs (ie, handwashing, proper coughing habits, social distancing, mask wearing); estimate the risks between the COVID-19 infection and the four NPIs; and understand potential risk compensating effects among the four NPIs in relation to the COVID-19 infection (eg, can wearing a mask further reduce the risk of infection among those who do and do not practice the other three NPIs?).

## Methods

### Study Sample

We conducted an online survey between February 22, 2020 (with total confirmed cases of 77,000 and daily cases of 1500), to March 5, 2020 (with a confirmed total case of 80,500 and daily cases of 151). Given that the whole of China was in lockdown during this period, it was almost impossible to conduct a random sample survey. We chose to conduct our study through the Chinese social media app Wechat (similar to “WhatsApp”) and Weibo (similar to “Twitter”). We adopted a snowballing sampling methodology through three social networks: (1) students and staff at Tongji Medical College and Chongqing Medical University; (2) Wanzhou District Centre for Disease Prevention and Control, Chongqing Municipality; and (3) the study team. The inclusion criteria were Chinese citizens who were currently living in Mainland China during the study period, having a mobile phone or computer, and willing to answer all questions. The exclusion criteria were those who did not consent to participate, those who did not answer all the questions, questionnaires completed in less than 2 minutes, and repeated questionnaires from the same Internet Protocol address. During the study period, the survey web page was browsed 21,673 times with a total of 8431 questionnaires returned. After excluding those illegible questionnaires and those who were younger than 18 years, the final study sample was 8158.

The Ethics Committee of Chongqing Medical University approved our study protocol. There was an introduction document before the study questionnaire that provided the respondents with the background, aims, and estimated time (10 minutes) for completing the survey. Respondents were asked for their agreement to participate in the study and to answer the questions faithfully, and were assured confidentiality, anonymity, and that no individual data would be disclosed. After the confirmation of their willingness to participate in the study voluntarily, the participants were directed to complete the online questionnaire. We plan to disseminate the results to study participants whenever appropriate.

### The Roles of the Funding Bodies

The funding bodies played no role in the study design, conduct, analysis, interpretation, and the decision to publish the results.

### Measures

A multidisciplinary team of 11 experts were involved in the development of the survey instrument. The team included two epidemiologists, two sociologists, one administrative specialist, one statistician, one psychologist, and four postgraduate students. The research team initially had serial meetings to decide the research aims, methodology, as well as the responsibilities of all team members, including their roles in the literature review and seeking ethics approval. The team then developed the survey instrument that included title, number, and content of all sections as well as every question within a section through an iterative process, which resulted in four major revisions of the first draft. The team also conducted two pilot tests. A group of 15 postgraduate students and staff members of the School of Public Health and Management of Chongqing Medical University participated in two testing pilots of the survey instrument (one test for the earlier version and one test for the near final version). As part of the pilot test, the group were asked a set of questions and then participated in an interview with one of the study team members. The interviews focused on both the survey questionnaires and the user-friendliness of its application in Webchat and Weibo. The discussions around the questionnaire were focused on the clarity of the questions, the readability of the questionnaire, the length of the survey, the overlapping and volume of questions among different sections, and the logic clarity between linked questions. The issues raised during the pilot testing were discussed during the team meetings, and necessary revisions were made. The final instrument included six sections and 79 questions in total, including (1) demographic information (16 questions), (2) knowledge and preventive behaviors related to COVID-19 (2 questions), (3) health status and related health behaviors (32 questions), (4) information source during the COVID-19 epidemic (7 questions), (5) perception and preparedness related to COVID-19 (15 questions), (6) satisfaction with the government’s performance in containing the COVID-19 epidemic (6 questions), and an open-ended question asking respondents to share their most important thoughts on the situation. A copy of the final questionnaire is attached (Multimedia Appendices 1 and 2).

The final questions included in this study were personal and family demographics including age, gender, location of residence, education, occupation, family monthly income, smoking habits during the last month (with over 100 cigarettes smoked over the lifetime), drinking alcohol during the last month, height and weight, been infected with COVID-19, marital status, if one of the family members is a health professional, the severity of the community infection where the respondent was living, and if one of the family members was part of local community efforts against COVID-19; perceived risk, attitude, information source, knowledge, and the four NPIs, and if the respondent had repeatedly used a mask; and self-isolation including if the respondents had a Chinese New Year party (2 days: January 24-25, 2020) with invited guests, the main reason a family member stayed home the longest, the main reason a family member went out often, the approach taken by the respondent when they went out (ie, shorten the time to avoid infection, as usual, stay longer given the restrictions, or uncertainty).

### Statistical Analysis

**Figure 1:**
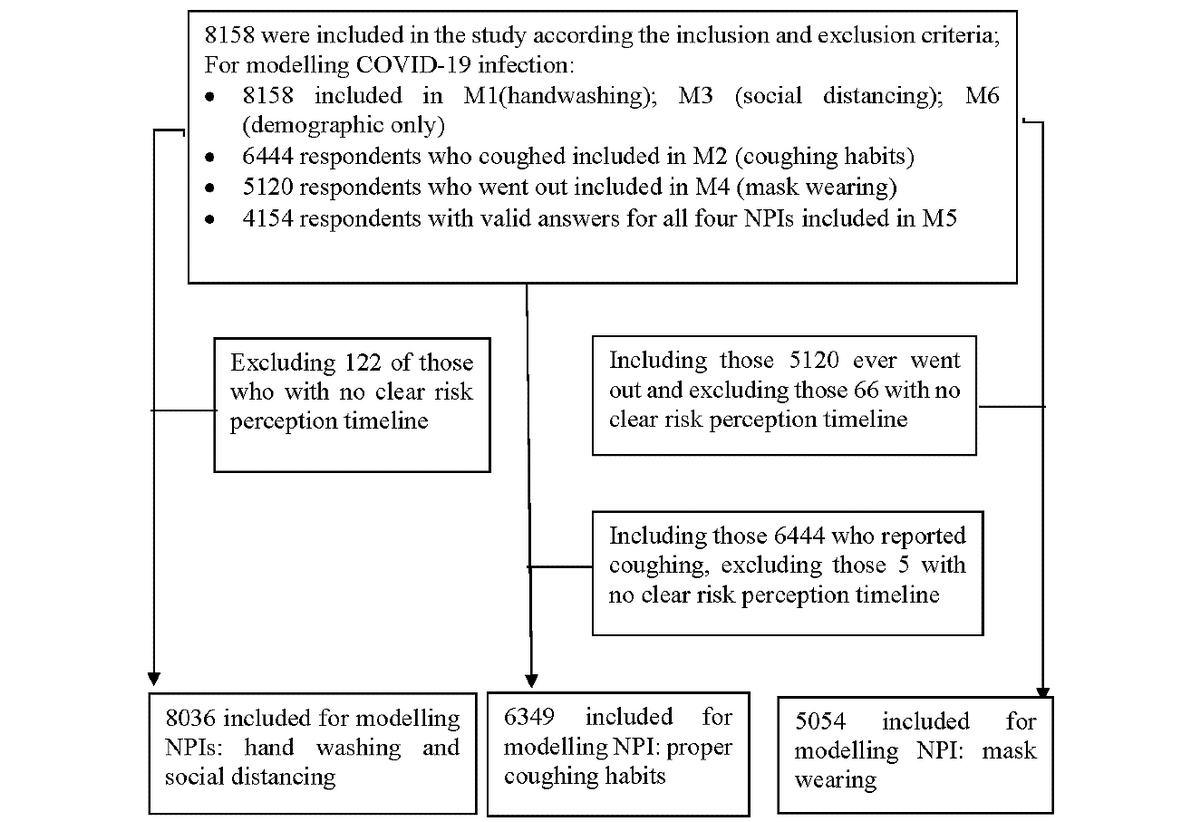
A flowchart of sample sizes for the modeling of the four NPIs (Multimedia Appendix 2: eTable 1) and COVID-19 infection (Tables 5 and 6). M: model; NPI: nonpharmaceutical intervention.

Frequencies of demographic, perceived risk, knowledge, attitude, and the four NPIs as well as self-isolation behaviors were described. The risks between the COVID-19 infection and four binary NPIs were tested using Fisher exact tests. The absolute risk difference, risk ratio (RR), and their 95% CIs were also presented. We modeled the four NPIs using logistic regression. The reasons that we also included proper coughing habit as an end point was that the habit may not only potentially reduce other people’s risk of developing a COVID-19 infection but may also reduce a person’s own risk through enhanced self-protection (eg, turning away from those who did not practice proper coughing habit) or through indirectly influencing other people’s coughing behavior as a role model. The predictors included the demographic characteristics, social economics status, family and social environment, perceived risk of situation, attitude (belief), and knowledge on the four NPIs. We explored the risk between a COVID-19 infection and the four NPIs using a similar approach but excluded knowledge, attitude, and risk perception of the four NPIs based on a penalized maximum likelihood function logistic regression [8,9], which provides consistent estimates in situations of sparse event and total separation. The modeling results for the four NPIs separately and combined (model 1-model 5) were compared to the results of the baseline model with only social demographic variables (model 6). RRs, odds ratios (ORs), and their 95% CIs were presented where appropriate. We explored the potential risk compensating effects among the four NPIs through a pairwise NPIs comparison of infection rates and through the comparison of infection rates of wearing a mask across a combination of the other three NPIs. A flowchart of different sample sizes for the modeling of the four NPIs and the COVID-19 infections are presented (Figure 1). The data management and statistical analysis were done through SPSS v25 (IBM Corp) and Stata v16 (StataCorp). *P* values less than .05 were considered as indicative of significance.

## Results

### Infection Rate of COVID-19 and Social Demographic of Respondents

In total, 8158 adults were included in the study, and 57 (0.7%) were infected with COVID-19. The respondents were predominantly female (n=5138, 63%), from younger age groups (18-39 years: n=5017, 62%), and living in the city (n=5833, 71.5%). Close to 45% of respondents had undergraduate and above education and close to one-quarter were students (Table 1). The family income from the previous month had a large range (¥0-¥4,000,000; a currency exchange rate of US $1=¥7 is applicable) with close to 20% of them less than ¥2000. Around 13% of respondents smoked and drank alcohol during the previous month. Over half of them had a normal BMI [10]. Close to 72% (n=5854) of respondents had a partner, and 54% (n=4420) were living with one. Close to 37% (n=3001) had a family member who was a health professional, and 35% (n=2835) had a family member who was part of the local community efforts against COVID-19. Over 54% (n=4439) were from the area outside Hubei Province, which had fewer than 100 cases; 42% (n=3400) from the areas outside Hubei Province, which had more than 100 infected cases; and 4% (n=319) from Hubei Province, the epicenter of the Chinese COVID-19 epidemic (Table 1).

**Table 1:** The demographic characteristics of the study sample.

Characteristics		Participants (N=8158), n (%)
**Age groups (years)**		
	18-39	5017 (61.5)
	40-59	2902 (35.6)
	≥60	239 (2.9)
Male (vs female)		3020 (37.0)
Currently living in city (vs rural area)		5833 (71.5)
**Education**		
	Primary school	130 (1.6)
	High school	2040 (25.0)
	Professional college	2331 (28.6)
	University/postgraduate	3657 (44.8)
**Occupation**		
	Health professionals	1373 (16.8)
	Government payee	1814 (22.2)
	Factory workers/managers	1485 (18.2)
	Farmers	313 (3.8)
	Students	2006 (24.6)
	Others	1167 (14.3)
**Family monthly income (¥)**		
	0-1000	607 (7.4)
	1001-2000	994 (12.2)
	2001-4000	1995 (24.5)
	4001-6000	1698 (20.8)
	6001-8000	714 (8.8)
	8001-10,000	977 (12.0)
	10,001-20,000	620 (7.6)
	20,001-4,000,000	216 (2.6)
	Not sure/unanswered	337 (4.1)
Smoked during the last month (yes)		1087 (13.3)
**Drinking during the last month**		
	Yes	1088 (13.3)
	Give up	308 (3.8)
	Do not drink	6762 (82.9)
**BMI**		
	Underweight	1574 (19.3)
	Normal	4263 (52.3)
	Overweight	1264 (15.5)
	Obese	133 (1.6)
	Not available	924 (11.3)
Infected with COVID-19		57 (0.7)
**Do you currently live with your partner?**		
	Yes	4420 (54.2)
	No	1434 (17.6)
	I do not have a partner	2304 (28.2)
Do you have a health professional family member? (yes)		3001 (36.8)
**From the area with community infection**		
	Hubei Province	319 (3.9)
	Outside Hubei Province with ≥100 cases	3400 (41.7)
	Other	4439 (54.4)
Is a family member part of local community efforts against COVID-19?		2835 (35.1)

### Risk Perception, Information Source, Knowledge, Attitude, the Four NPIs, and Self-Isolation

Of the 8158 respondents, close to 7% (n=561) of respondents were aware of the situation’s seriousness on January 11, 2020, when the first COVID-19–related patient death was announced by the Wuhan Health Commission; 39% (n=3182) on January 20, 2020, with the announcement of COVID-19 transmission among humans; 29% (n=2354) on January 23, 2020, with the lockdown of Wuhan City; and 24% (n=1562) after January 24, 2020, due to the activation of the level 1 public emergency responding scheme by local governments as well as strict measures and lockdown of neighborhood or villages [11]. Only 9 (0.1%) respondents did not think it was serious at the time (Table 2). Overwhelmingly, the majority (n=8094, 99.2%) strongly agreed with the position that the fight against COVID-19 was everyone’s responsibility (Table 3). Close to 97% perceived government websites, apps, and the public media as the most authoritative sources of information; 90% (n=7396) felt that government websites, apps, and the public media were also the most involved source of information; 99.6% (n=8129) of respondents knew why and how to wash hands properly during the COVID-19 outbreak period; 97.2% (n=7927) were aware of the proper procedures when coughing (turning away from people and covering mouth and nose when coughing and washing hands afterwards); 97.8% (n=7975) knew the right way of practicing social distancing (ie, keeping social distance more than 1 meter and avoiding close contact with those who had a fever or cough); and 99.9% (n=8146) knew why and how to wear a mask. The overwhelming majority also reported that they translated this knowledge into practice: 96.8% (n=7895) when washing hands, 93.1% (n=5997/6444) when coughing, 87.1% (n=7104) when social distancing, and 97.9% (5012/5120) when wearing a mask (Table 3).

**Table 2:** When participants felt the situation was serious.

Events	Participants (N=8158), n
Announcement of first death of patients with COVID-19 on January 13, 2020	561
Announcement of COVID-19 transmission among humans on January 19, 2020	3182
Lockdown of Wuhan City on January 23, 2020	2354
Activation of the public emergency responding scheme by local government on January 24, 2020	1562
Strict lockdown of neighborhood after January 24, 2020	377
Other critical events	113
Never felt it was serious	9

Our multivariable models found that the knowledge of the relevant NPIs was one of the strongest predictors of that behavior (OR 22.6 for handwashing, OR 4.26 for social distancing; all *P*<.001), and positive knowledge level associated with a proper coughing habit and mask wearing were at 100% and were excluded from the models (Multimedia Appendix 3, first eTable). The belief that the task of fighting against COVID-19 is everyone’s responsibility was positively associated with handwashing (OR 5.59, *P*<.001), social distancing (OR 3.76, *P*<.001), and mask wearing (OR 26.89, *P*<.001). Those who perceived the seriousness of the situation before the lockdown of Wuhan City were more likely to practice handwashing (OR 1.43, 95% CI 1.08-1.90; *P*=.01) and a proper coughing habit (OR 1.54, 95% CI 1.24-1.92; *P*<.001). Those who had a family member involved in the local community efforts against COVID-19 and people from outside the Hubei Province were more likely associated with positive NPIs. In comparison to those who were currently living with a partner, those who did not have partner were less likely to practice handwashing (OR 0.57, 95% CI 0.35-0.95; *P*=.03) and proper coughing habits (OR 0.59, 95% CI 0.41-0.83; *P*=.003) but were more likely to practice social distancing (OR 1.64; *P*<.001). The respondents who had a normal body weight were more likely to practice social distancing (OR 1.21, 95% CI 1.01-1.46; *P*=.04) than those who were underweight. Nonsmokers were more likely to practice social distancing (OR 1.39, 95% CI 1.14-1.71; *P*=.001) than smokers. Family income, education, occupation, residential area, sex, and age groups demonstrated differential impacts on different NPIs (Multimedia Appendix 3, first eTable).

Approximately 23% of respondents reported they had a Chinese New Year party with invited guests (Multimedia Appendix 3, second eTable). Out of 8158 respondents, the major reasons for the family member who stayed longest at home included complying with the call from the government (n=5354, 65.6%), self- or compulsory isolation (n=1107, 13.6%), fear of the virus (n=469, 5.7%), the focus on family protection (n=310, 3.8%), and no mask (n=242, 3.0%). The main reasons for going out, for 5120 respondents, were shopping (n=2073, 40.5%), partaking in work related to controlling COVID-19 (n=1643, 32.1%), usual employment (n=1098, 21.4%), going for a walk (n=131, 2.6%), receiving delivery (n=38, 0.7%), and socializing and dinner parties (n=17, 0.3%). Over 74% shortened the time to avoid infection when they were out; close to 20% acted in the usual way; and 1% (n=50) stayed longer than usual, given the restrictions and difficulties to leave home (Multimedia Appendix 3 second eTable).

**Table 3:** Attitude, source of information, knowledge, and the four nonpharmaceutical interventions.

Item		Participant (N=8158), n (%)
Agree with that the fight with COVID-19 is everyone’s job		8094 (99.2)
**Perceived most authoritative source of information**		
	Government websites/app/public media	7902 (96.9)
	Weibo/Webchat friends	128 (1.6)
	QQ/webchat groups	64 (0.8)
	Family/friends	40 (0.5)
	Other	24 (0.3)
**Perceived most interested source of information**		
	Government websites/app/public media	7396 (90.7)
	Weibo/Webchat friends	470 (5.8)
	QQ/webchat groups	218 (2.7)
	Family/friends	60 (0.7)
	Other	14 (0.2)
Know why and how to wash hands		8129 (99.6)
Know the proper habit when coughing		7927 (97.2)
Know why and how to practice social distancing		7975 (97.8)
Know why and how to wear a mask in public		8146 (99.9)
Washing hands (yes)		7895 (96.8)
Acting in proper habit when coughing^a^		5997 (93.1)
Practicing social distancing		7104 (87.1)
Wearing a mask when going out^b^		5012 (97.9)
Repeated use of a mask^b^		5117 (99.9)

^a^Only 6444 included as 1714 person reported that they did not cough during the last month.

^b^Only the 5120 respondents that went out during the period after the outbreak were included.

### Risk Association Between a COVID-19 Infection and Handwashing, Coughing Habits, Social Distancing, and Mask Wearing

The distribution of the COVID-19 infection across demographic and social economic status variables are presented (Table 4). The bivariate analyses between individual NPIs and the COVID-19 infection showed that there was a significantly increased risk of COVID-19 infection (Table 5) for those who did not wash their hands (2.28% vs 0.65%; RR 3.53, 95% CI 1.53-8.15; *P*=.009), who did not practice proper coughing (1.79% vs 0.73%; RR 2.44, 95% CI 1.15-5.15; *P*=.03), who did not practice social distancing (1.52% vs 0.58%; RR 2.63, 95% CI 1.48-4.67; *P*=.002), and who did not wear a mask (7.41% vs 0.6%; RR 12.38, 95% CI 5.81-26.36; *P*<.001). The adjusted ORs were 4.67 (95% CI 1.86-11.74; *P*=.001) for not washing hands, 2.78 (95% CI 1.22-6.33; *P*=.02) for not practicing proper coughing, 2.13 (95% CI 1.17-3.85; *P*=.01) for not practicing social distancing, and 11.03 (95% CI 4.53-26.84; *P*<.001) for not wearing a mask (Tables 6 and 7, model 1-model 4). The model that adjusted all four NPIs plus social demographic variables (Table 7, model 5) showed that not wearing a mask was the only significant predictor of infection (OR 7.20, 95% CI 2.24-23.11; *P*<.001). In comparison with those who were only primary school educated, those with a high school qualification showed they were less likely to be infected (OR 0.12, 95% CI 0.05-0.31; *P*<.001). This was similar to those who had professional college qualifications (OR 0.10, 95% CI 0.03-0.29; *P*<.001) or university degrees (OR 0.15, 95% CI 0.05-0.46; *P*=.001; Table 7, model 6). Nonsmokers were less likely to be infected than smokers (OR 0.40, 95% CI 0.20-0.80; *P*=.01), and those with a monthly family income of ¥8001-¥10,000 were less likely to be infected than those having a monthly family income of less than ¥1000 (OR 0.20, 95% CI 0.05-0.88; *P*=.03; Table 7, model 6). Consisting of the effective sizes of the four NPIs from both bivariable and multivariable analyses, the areas under the receiver operating characteristics curve (AUROCs) were 0.749, 0.769, 0.749, and 0.825 for handwashing, coughing habits, social distancing, and mask wearing, respectively (Tables 6 and 7, model 1-model 4). The AUROC values demonstrated a fair predictive power for the handwashing, coughing habits, and social distancing models but good predictive power of the mask wearing multivariable model.

**Table 4:** The rate of COVID-19 infection (57/8158, 0.7%) across social demographic variables.

Variables		Participants, n	Participants infected with COVID-19, n (%)	*P* value^a^
**Age groups (years)**				.14
	18-39	5017	29 (0.58)	
	40-59	2902	25 (0.86)	
	≥60	239	3 (1.26)	
**Sex**				.008
	Male	3020	31 (1.03)	
	Female	5138	26 (0.51)	
**The area that you are currently living**				.46
	Rural	2325	19 (0.82)	
	City	5833	38 (0.65)	
**Education**				<.001
	Primary school	130	8 (6.15)	
	High school	2040	14 (0.69)	
	Professional college	2331	13 (0.56)	
	University/postgraduate	3657	22 (0.60)	
**Occupation**				.17
	Health professionals	1373	9 (0.66)	
	Government payee	1814	14 (0.77)	
	Factory workers/managers	1485	14 (0.94)	
	Farmers	313	4 (1.28)	
	Students	2006	7 (0.35)	
	Others	1167	9 (0.77)	
**Income (¥)**				.20
	0-1000	607	8 (1.32)	
	1001-2000	994	7 (0.70)	
	2001-4000	1995	16 (0.80)	
	4001-6000	1698	13 (0.77)	
	6001-8000	714	4 (0.56)	
	8001-10,000	977	2 (0.20)	
	10,001-20,000	620	5 (0.81)	
	20,001-4,000,000	216	2 (0.93)	
	Not sure/unanswered	337	0 (0.00)	
**Smoked during the last month**				<.001
	Yes	1087	20 (1.84)	
	No	7071	37 (0.52)	
**Drank alcohol during the last month**				.002
	Yes	1088	12 (1.10)	
	Gave up	308	7 (2.27)	
	Do not drink	6762	38 (0.56)	
**BMI**				.85
	Underweight	1574	12 (0.76)	
	Normal	4263	29 (0.68)	
	Overweight	1264	7 (0.55)	
	Obesity	133	1 (0.75)	
	Not available	924	8 (0.87)	
**Currently live with your partner?**				.01
	Yes	4420	36 (0.81)	
	No	1434	14 (0.98)	
	I do not have a partner	2304	7 (0.30)	
**Family member who is a health professional**				.17
	Yes	3001	26 (0.87)	
	No	5157	31 (0.60)	
**From the area with community infection**				.60
	Hubei Province	319	3 (0.94)	
	Outside Hubei province with ≥100 cases	3400	21 (0.62)	
	Other	4439	33 (0.74)	
**Family member who is part of local community efforts against COVID-19**				.17
	Yes	2865	25 (0.87)	
	No	5293	32 (0.60)	

^a^*P* values from Fisher exact test.

**Table 5:** The association between COVID-19 infection and four nonpharmaceutical interventions.

Nonpharmaceutical interventions		Risk of COVID-19 infection, n (%)	Risk difference (95% CI), %	Risk ratio (95% CI)	*P* value^a^
**Washing hands (N=8158)**			1.63 (–0.18 to 3.45)	3.53 (1.53 to 8.15)	.009
	No (n=263)	6 (2.28)			
	Yes (n=7895)	51 (0.65)			
**Acting properly when coughing (n=6444)**			1.06 (–0.19 to 2.3)	2.44 (1.15 to 5.15)	.03
	No (n=447)	8 (1.79)			
	Yes (n=5997)	44 (0.73)			
**Practicing social distancing (N=8158)**			0.94 (0.18 to 1.7)	2.63 (1.48 to 4.67)	.002
	No (n=1054)	16 (1.52)			
	Yes (n=7104)	41 (0.58)			
**Wearing a mask outdoors (n=5120)**			6.81 (1.87 to 11.75)	12.38 (5.81 to 26.36)	<.001
	No (n=108)	8 (7.41)			
	Yes (n=5012)	30 (0.60)			

^a^*P* values from Fisher exact test.

**Table 6:** Penalized logistic regression model results for COVID-19 infection and handwashing, coughing habits, social distancing, and mask wearing (models 1-3).^a^

Variables		Infected (model 1; N=8158), OR^b^ (950% CI)	Infected (model 2; n=6444), OR (95% CI)	Infected (model 3; N=8158), OR (95% CI)
**Age groups (years), reference: 18-39 years**				
	40-59	0.97 (0.51-1.85)	0.88 (0.45-1.73)	0.93 (0.49-1.78)
	≥60	1.55 (0.46-5.25)	1.68 (0.49-5.83)	1.71 (0.51-5.75)
Sex (female vs male)		0.76 (0.39-1.47)	0.72 (0.36-1.44)	0.76 (0.39-1.48)
Living in city (urban vs rural)		0.94 (0.50-1.77)	0.81 (0.42-1.56)	0.91 (0.48-1.71)
**Education, reference: primary school**				
	High school	0.13 (0.05-0.33)***	0.12 (0.04-0.34)***	0.12 (0.05-0.31)***
	Professional college	0.10 (0.03-0.30)***	0.10 (0.03-0.31)***	0.10 (0.03-0.29)***
	University/postgraduate	0.16 (0.05-0.48)**	0.14 (0.04-0.46)**	0.16 (0.05-0.48)**
**Occupation, reference: health professionals**				
	Government payee	1.51 (0.63-3.67)	1.67 (0.65-4.28)	1.54 (0.63-3.73)
	Factory workers/managers	1.82 (0.66-5.00)	2.12 (0.74-6.09)	1.87 (0.68-5.17)
	Farmers	1.02 (0.25-4.14)	0.95 (0.21-4.39)	0.99 (0.24-4.13)
	Students	0.97 (0.26-3.69)	0.90 (0.22-3.70)	1.10 (0.29-4.17)
	Others	1.63 (0.51-5.19)	1.79 (0.52-6.09)	1.73 (0.54-5.55)
**Family monthly income (¥), reference: ¥0-¥1000**				
	1001-2000	0.70 (0.25-1.96)	0.58 (0.20-1.75)	0.61 (0.22-1.68)
	2001-4000	0.79 (0.32-1.93)	0.69 (0.27-1.74)	0.73 (0.30-1.77)
	4001-6000	0.68 (0.27-1.71)	0.62 (0.24-1.62)	0.63 (0.25-1.57)
	6001-8000	0.54 (0.16-1.83)	0.56 (0.16-1.93)	0.50 (0.15-1.69)
	8001-10,000	0.21 (0.05-0.93)*	0.13 (0.02-0.76)*	0.20 (0.05-0.88)*
	10,001-20,000	0.67 (0.20-2.22)	0.62 (0.19-2.07)	0.63 (0.20-2.06)
	20,001-4,000,000	0.54 (0.12-2.51)	0.53 (0.11-2.48)	0.55 (0.12-2.45)
	Not sure/unanswered	0.15 (0.01-2.63)	0.15 (0.01-2.62)	0.13 (0.01-2.32)
Smoked during the last month (no vs yes)		0.38 (0.19-0.76)**	0.38 (0.18-0.77)**	0.42 (0.21-0.84)*
**Drank alcohol during the last month, reference: yes**				
	Gave up	1.96 (0.76-5.04)	2.07 (0.79-5.44)	1.94 (0.75-4.97)
	Do not drink	0.88 (0.42-1.87)	0.87 (0.40-1.89)	0.84 (0.40-1.78)
**BMI, reference: underweight**				
	Normal	0.80 (0.41-1.59)	0.76 (0.37-1.57)	0.80 (0.41-1.59)
	Overweight	0.49 (0.19-1.25)	0.55 (0.21-1.43)	0.49 (0.19-1.26)
	Obese	0.88 (0.16-4.94)	1.06 (0.19-6.06)	0.94 (0.17-5.28)
	Not available	1.15 (0.46-2.86)	1.17 (0.45-3.07)	1.11 (0.45-2.78)
**Currently live with your partner, reference: yes**				
	No	1.34 (0.69-2.63)	1.36 (0.67-2.77)	1.37 (0.70-2.69)
	I do not have a partner	0.50 (0.19-1.34)	0.71 (0.26-1.90)	0.55 (0.21-1.49)
Family member who is health professional (yes vs no)		0.53 (0.26-1.08)	0.59 (0.28-1.23)	0.50 (0.24-1.01)
**Living in the area with a community infection, reference: Hubei Province**				
	Outside Hubei Province with ≥100 cases	0.46 (0.14-1.53)	0.46 (0.13-1.61)	0.49 (0.14-1.65)
	Other	0.74 (0.23-2.36)	0.82 (0.25-2.63)	0.81 (0.25-2.62)
Family member who is part of the local community efforts against COVID-19 (no vs yes)		0.70 (0.37-1.30)	0.64 (0.34-1.22)	0.76 (0.41-1.42)
Hand washing (no vs yes)		4.67 (1.86-11.74)**	N/A^c^	N/A
Proper coughing habit (no vs yes)		N/A	2.78 (1.22-6.33)*	N/A
Social distancing (no vs yes)		N/A	N/A	2.13 (1.17-3.85)*
Mask wearing (no vs yes)		N/A	N/A	N/A
Constant		0.67 (0.09-4.81)	0.90 (0.11-6.99)	0.60 (0.08-4.42)
AUROC^d^		0.749	0.769	0.749

^a^Models 1-5 are models adjusting for social demographic variables plus handwashing, coughing habits, social distancing, mask wearing, and all four nonpharmaceutical interventions together, respectively. Model 6 is adjusting for social demographic variables only. For each model, the convergency of penalized maximum likelihood function was monitored, and coefficients and their 95% CIs were examined. Each model converged normally in a short period and no irregularity of coefficients and standard errors were identified.

^b^OR: odds ratio.

^c^N/A: not applicable.

^d^AUROC: area under the receiver operating characteristic curve.

*P<.05, **P<.01, ***P<.001

**Table 7:** Penalized logistic regression model results for COVID-19 infection and handwashing, coughing habits, social distancing, and mask wearing.^a^

Variables		Infected (model 4; n=5120), OR^b^ (95% CI)	Infected (model 5; n=4154), OR (95% CI)	Infected (model 5; N=8,158), OR (95% CI)
**Age groups (years), reference: 18-39 years**				
	40-59	1.07 (0.47-2.42)	1.17 (0.49-2.81)	0.95 (0.50-1.81)
	≥60	3.16 (0.82-12.11)	3.79 (0.96-14.94)	1.68 (0.50-5.71)
Sex (female vs male)		0.65 (0.29-1.49)	0.69 (0.29-1.66)	0.73 (0.38-1.42)
Living in city (urban vs rural)		0.85 (0.37-1.95)	0.75 (0.32-1.77)	0.89 (0.47-1.68)
**Education, reference: primary school**				
	High school	0.09 (0.03-0.30)***	0.11 (0.03-0.43)**	0.12 (0.05-0.31)***
	Professional college	0.09 (0.03-0.34)***	0.11 (0.03-0.47)**	0.10 (0.03-0.29)***
	University/postgraduate	0.08 (0.02-0.33)***	0.10 (0.02-0.47)**	0.15 (0.05-0.46)***
**Occupation, reference: health professionals**				
	Government payee	1.74 (0.60-5.06)	1.59 (0.52-4.85)	1.57 (0.65-3.81)
	Factory workers/managers	1.97 (0.56-6.89)	1.80 (0.49-6.55)	1.95 (0.71-5.33)
	Farmers	1.35 (0.25-7.27)	0.76 (0.12-4.97)	1.08 (0.26-4.43)
	Students	1.00 (0.19-5.36)	0.69 (0.12-4.03)	1.18 (0.31-4.43)
	Others	2.02 (0.49-8.27)	2.17 (0.52-9.04)	1.72 (0.54-5.51)
**Family monthly income (¥), reference: ¥0-¥1000**				
	1001-2000	0.42 (0.11-1.57)	0.58 (0.14-2.38)	0.62 (0.23-1.72)
	2001-4000	0.77 (0.26-2.28)	0.90 (0.28-2.92)	0.73 (0.30-1.78)
	4001-6000	0.47 (0.14-1.52)	0.64 (0.18-2.25)	0.63 (0.25-1.58)
	6001-8000	0.56 (0.13-2.37)	0.73 (0.17-3.25)	0.50 (0.15-1.68)
	8001-10,000	0.31 (0.06-1.49)	0.24 (0.03-1.62)	0.20 (0.05-0.88)*
	10,001-20,000	0.43 (0.10-1.87)	0.53 (0.11-2.49)	0.62 (0.19-2.03)
	20,001-4,000,000	0.24 (0.02-2.61)	0.21 (0.01-3.85)	0.60 (0.14-2.64)
	Not sure/unanswered	0.11 (0.00-2.75)	0.26 (0.01-4.97)	0.13 (0.01-2.36)
Smoked during the last month (no vs yes)		0.67 (0.29-1.58)	0.65 (0.27-1.57)	0.40 (0.20-0.80)**
**Drank alcohol during the last month, reference: yes**				
	Gave up	1.29 (0.43-3.87)	1.34 (0.43-4.20)	2.00 (0.78-5.10)
	Do not drink	0.48 (0.20-1.13)	0.47 (0.19-1.15)	0.85 (0.40-1.80)
**BMI, reference: underweight**				
	Normal	0.56 (0.25-1.25)	0.57 (0.24-1.33)	0.79 (0.40-1.57)
	Overweight	0.26 (0.07-0.90)*	0.28 (0.08-0.99)*	0.48 (0.19-1.25)
	Obese	0.31 (0.02-5.81)	0.38 (0.02-7.45)	0.88 (0.16-4.97)
	Not available	1.25 (0.46-3.44)	1.17 (0.40-3.46)	1.13 (0.45-2.82)
**Currently living with a partner, reference: yes**				
	No	1.62 (0.69-3.82)	1.28 (0.50-3.26)	1.38 (0.70-2.69)
	I don’t have a partner	0.98 (0.30-3.20)	1.19 (0.36-3.91)	0.53 (0.20-1.41)
Family member who is a health professional (yes vs no)		0.51 (0.22-1.19)	0.61 (0.25-1.48)	0.50 (0.24-1.01)
**Living in an area with community infection, reference: Hubei Province**				
	Outside Hubei Province with ≥100 cases	0.24 (0.06-0.95)*	0.25 (0.06-1.09)	0.45 (0.13-1.51)
	Other	0.68 (0.19-2.50)	0.74 (0.20-2.81)	0.78 (0.25-2.47)
Family member who is part of the local community efforts against COVID-19 (no vs yes)		0.51 (0.24-1.09)	0.46 (0.21-1.04)	0.74 (0.40-1.37)
Handwashing (no vs yes)		N/A^c^	1.82 (0.40-8.32)	N/A
Proper coughing habit (no vs yes)		N/A	1.88 (0.60-5.94)	N/A
Social distancing (no vs yes)		N/A	1.07 (0.46-2.46)	N/A
Mask wearing (no vs yes)		11.03 (4.53-26.84)***	7.20 (2.24-23.11)***	N/A
Constant		2.04 (0.19-21.47)	1.64 (0.13-20.42)	0.78 (0.11-5.62)
AUROC^d^		0.825	0.823	0.748

^a^Models 1-5 are models adjusting for social demographic variables plus handwashing, coughing habits, social distancing, mask wearing, and all four nonpharmaceutical interventions together, respectively; model 6 is adjusting for social demographic variables only. For each model, the convergency of penalized maximum likelihood function was monitored, and coefficients and their 95% CIs were examined. Each model converged normally in a short time and no irregularity of coefficients and standard errors was identified.

^b^OR: odds ratio.

^c^N/A: not applicable.

^d^AUROC: area under the receiver operating characteristic curve.

*P<.05, **P<.01, ***P<.001

### Potential Risk Compensating Effects Among the Four NPIs Against a COVID-19 Infection

**Figure 2:**
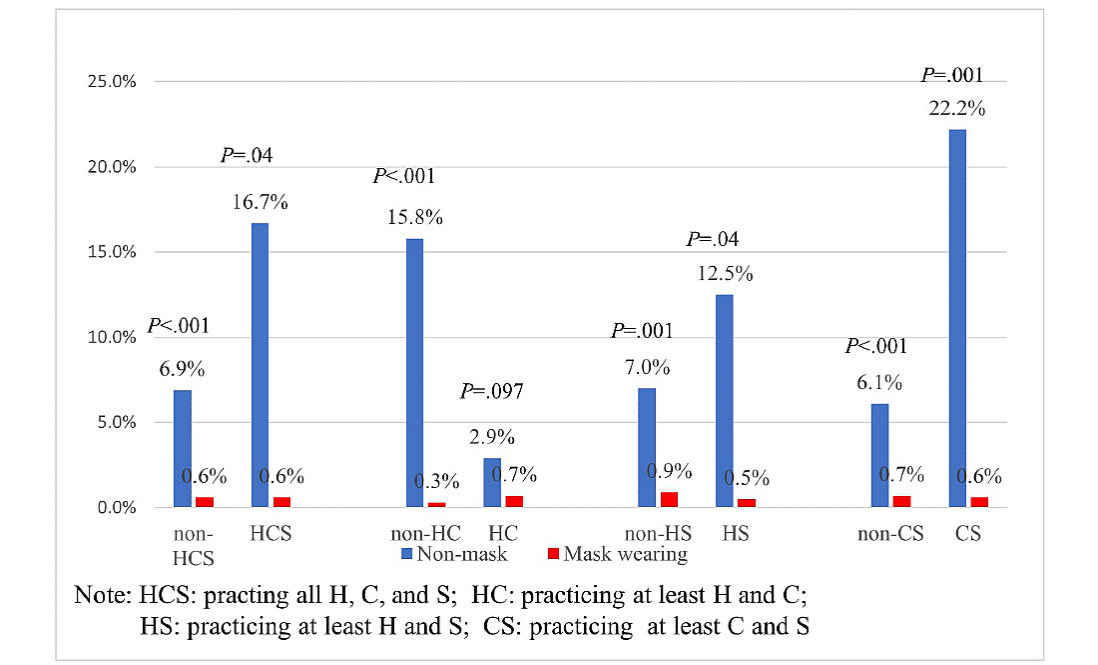
The COVID-19 infection rates between mask wearing and the combinations of the other three nonpharmaceutical interventions: H, C, and S. *P* values from Fisher exact test. C: proper coughing habit; H: handwashing; S: social distancing.

The pairwise distributions of the COVID-19 infection rate among the four NPIs are presented (Table 8). Wearing a mask (vs not) was associated with a significantly reduced risk of COVID-19 infection among those who practiced handwashing (0.6% vs 5.3%; RR 0.11; *P*<.001), proper coughing (0.7% vs 3.9%; RR 0.18; *P*=.02), and social distancing (0.5% vs 16.7%; RR 0.03; *P*=.002). Handwashing showed a trend toward a further reduced risk of infection for those who did not practice social distancing (RR 0.25; *P*=.05). Among those who did not practice social distancing, those who had proper coughing habits were associated with a reduced risk of infection compared to those who did not have proper coughing habits (1.3% vs 4.4%; RR 0.29; *P*=.048). The added potential protection effect of mask wearing on different combinations of the other three NPIs are presented in Figure 2. For those who did practice all three NPIs (ie, handwashing, proper coughing, and social distancing), wearing a mask was associated with a significantly reduced risk of infection compared to those who did not (0.6% vs 16.7%; *P*=.04). Similarly, for those who did not practice all other three NPIs, wearing a mask was also associated with a significantly reduced risk of infection compared to not wearing a mask (Figure 2).

**Table 8:** The COVID-19 infection rates (%) and potential pairwise risk compensating effect among the four nonpharmaceutical interventions with RR and *P* values from exact tests.

Nonpharmaceutical interventions		Participants, n (%)	RR^a^ (95% CI)	*P* value
**Handwashing (no)**			0.03 (0.003-0.26)	.002
	Mask wearing (no)	13 (23.1)		
	Mask wearing (yes)	147 (0.7)		
**Handwashing (yes)**			0.11 (0.04-0.29)	<.001
	Mask wearing (no)	95 (5.3)		
	Mask wearing (yes)	4865 (0.6)		
**Proper coughing (no)**			0.02(0.004-0.08)	<.001
	Mask wearing (no)	11 (36.4)		
	Mask wearing (yes)	318 (0.6)		
**Proper coughing (yes)**			0.18 (0.05-0.57)	.02
	Mask wearing (no)	76 (3.9)		
	Mask wearing (yes)	3749 (0.7)		
**Social distancing (no)**			0.17 (0.06-0.45)	.002
	Mask wearing (no)	96 (6.3)		
	Mask wearing (yes)	958 (1.0)		
**Social distancing (yes)**			0.03 (0.01-0.11)	.002
	Mask wearing (no)	12 (16.7)		
	Mask wearing (yes)	4054 (0.5)		
**Proper coughing (no)**			0.33 (0.08-1.35)	.13
	Handwashing (no)	74 (4.1)		
	Handwashing (yes)	373 (1.3)		
**Proper coughing (yes)**			0.42 (0.10-1.72)	.21
	Handwashing (no)	118 (1.7)		
	Handwashing (yes)	5879 (0.7)		
**Social distancing (no)**			0.25 (0.73-0.86)	.05
	Handwashing (no)	58 (5.2)		
	Handwashing (yes)	996 (1.3)		
**Social distancing (yes)**			0.38 (0.12-1.21)	.11
	Handwashing (no)	205 (1.5)		
	Handwashing (yes)	6899 (0.6)		
**Social distancing (no)**			0.29(0.09-0.91)	.048
	Proper coughing (no)	90 (4.4)		
	Proper coughing (yes)	776 (1.3)		
**Social distancing (yes)**			0.58 (0.21-1.63)	.30
	Proper coughing (no)	357 (1.1)		
	Proper coughing (yes)	5221 (0.7)		

^a^RR: risk ratio.

## Discussion

To the best of our knowledge, our study is the first comprehensive report on the COVID-19 infection rate, perceived risk, knowledge, attitude, the four NPIs, as well as the self-isolation of a nationwide adult sample amid the late period of the COVID-19 epidemic in China. We found that most respondents were aware of the outbreak’s seriousness at different time periods and believed that it was everyone’s responsibility to fight the spread of COVID-19. The positive attitude, earlier risk perception, and relevant knowledge were among the strongest predictors of handwashing, proper coughing habits, social distancing, and mask wearing. Different social demographic factors also contributed to different NPIs. Those who were only primary school educated, had little family income, and were a smoker were associated with an increased risk of COVID-19 infection. Mask wearing, among the four practices, was the most important protection factor against a COVID-19 infection with an added preventive effect among those who practiced all or part of the other three NPIs.

Our findings of high knowledge levels among the Chinese public were consistent with the results of a previous study [12]. However, the previous online survey study was conducted at a much earlier stage (ie, January 27 to February 1, 2020) with a smaller sample, and over half of the respondents were from Hubei Province. In addition, it did not include the knowledge and behaviors of handwashing, proper coughing habit, social distancing, and self-isolation (but mask wearing was included). The widespread use of mobile phones, the internet, and social media apps such as Wechat (with an estimated 1.1 billion registered accounts in China in 2019 [13]) have significantly increased the speed and scope of information transmission in China and may be instrumental in the dissemination of COVID-19–related knowledge and information. This was also helped by the fact that over 90% of respondents believed that the government websites, public media, and apps were the most authoritative and involved sources of information for COVID-19. Our results suggested that respondents had an extremely positive attitude that fighting against COVID-19 was everyone’s responsibility, and its association with positive NPIs highlighted the success of a nationwide campaign in instilling the concept that everyone can, and should, make necessary contributions toward the fight against COVID-19.

Our study’s finding that the early perceived seriousness of the situation was a strong predicting factor for the use of NPIs reinforces the importance of transparency and timely dissemination of critical information regarding the COVID-19 pandemic. Our study shows that around 22% of respondents had a party during Chinese New Year (January 24-25, 2020), which is an important Chinese tradition. This period was immediately after the lockdown of Wuhan City (January 23), where there was confusion for those who lived outside the Hubei Province where there was no social distancing and self-isolation in place at the time. Such gatherings could have been avoided if the public had been equipped with real-time knowledge of the danger and seriousness of the situation. Evidence also supported the concept that the early swift NPIs by governments and the education of the public regarding the seriousness of the situation were critical in slowing the spread and flattening the curve [14]. Our study demonstrates that family influences (particularly those with health professionals and with someone being part of a community team fighting against COVID-19) could have a significant positive impact on individual’s behaviors. Respondents with various demographic characteristics (eg, age groups, sex, marital status, education, occupation, and smoker) had exhibited different NPIs. These findings may provide further opportunities for developing tailored health education campaigns and health policy interventions on segments of the population to maximize the effects of the NPIs. For example, specific policies and education could target smokers and the younger population to encourage certain behaviors such as proper coughing habit and the older population on other behaviors (eg, self-isolation, social distancing, and mask wearing).

The reported COVID-19 infection rate (ie, 57/8158, 0.73%) in our study is higher than the China national infection rate on March 5, 2020 (ie, 0.0056%), that reflected the fact that the disproportional sample were from the higher risk Hubei province. Our study found almost universal acceptance of the importance of mask wearing, and a high proportion (5012/5120, 97.9%) wore a mask in public after the outbreak. Our study found that mask wearing, among the four personal NPIs, was the most important protective measure against a COVID-19 infection. This may have policy implications. The Chinese public accepted the concept of wearing a mask possibly due to factors such as the previous severe acute respiratory syndrome epidemic experience [15], the coordinated nationwide education campaign, the earlier recognition of the existence of asymptomatic virus carriers, the strict measures in reinforcing such a role (eg, in shopping centers and public transportation), and the coordinated efforts in rationing the supply of masks to families over the shortage period. The necessity in wearing a mask in public may be controversial in different countries and agencies [16-20] despite the positive evidence in favoring wearing a mask in a simulated environment [21,22]. It is likely to be an evolving policy option depending on several factors including the availability of masks and fair distribution channels among society. Our findings that wearing a mask had an added preventive effect, even among those who did practice all or part of the other three NPIs, provided contradictive evidence regarding the opinion that the other three NPIs alone were sufficient in preventing the COVID-19 infection. It also did not support the opinion that wearing a mask could even increase the risk of infection through more facial contact. For those countries still in the grip of the pandemic or who are considering reopening their economy, a policy of encouraging or requiring the public to wear a mask may have a positive impact, especially in highly populated areas or in settings where other NPIs are difficult to implement (eg, in a bus, airplane, or shopping center). During the study period, there were still 3% (242/8158) of respondents who reported that *no mask* was the main reason for stopping them from going out, and most respondents had repeatedly used the same mask. Given the likelihood of a surge in demand for masks during an outbreak, public health agencies and related authorities may also need to provide practical and evidence-based guidance on when and how to appropriately reuse a mask. China contributed over half of the global mask manufacturing output before the outbreak but still faced the shortage of masks over the epidemic period [23]. It is important for governments and international agencies to rethink the adequacy of, and better approaches toward, their strategic stockpiles of masks and other personal protection equipment for the current and future pandemics.

Our study has several strengths. First, it was the largest study of its kind to cover the most critical period of the COVID-19 outbreak in China. Second, our study design and analysis was driven by policy needs and included many factors such as demographics, social economic status, family contextual factors, risk perception, knowledge, attitude, and personal practices. Third, the adoption of the internet survey methodology enabled us to complete our study in a critical period and in a cost-effective manner. Our study also has several limitations. First, our study sample had disproportionately more female, well-educated, and less smoker respondents, reflecting a typically young and healthy cohort in similar surveys. Thus, the frequencies of desirable knowledge levels and health behaviors may be overestimated, while less desirable outcomes such as lower family monthly income may be underestimated. However, the modeling results may be less susceptible to these potential biases. Second, our study results were from a particular period of the outbreak, and most of the respondents were from outside Hubei Province. The generalization of the results to other settings and countries may be limited. Third, our study was a cross-sectional population survey, and the association found between the predictors and outcomes should be interpreted with caution, and further intervention studies are needed in confirming our findings. Fourth, despite the relatively large sample size, the total cases of COVID-19 infections were still small so that the relationship between NPIs and a COVID-19 infection should be confirmed by other larger epidemiological studies. Fifth, the potential risk compensating effects of wearing a mask against other NPIs should be considered as being of a hypothesis-generating nature given the potential limitations previously outlined. Sixth, all the information collected in the study was self-reported, which could have potential biases. Common to any observational studies with multiple outcomes and modeled with different effective sample sizes, the interpretations and generalization of the results should be strictly limited to the same setting and be aware of multiple tests risks.

In summary, our study found a high level of risk perception; positive attitude; desirable knowledge; and practices in handwashing, proper coughing habit, social distancing, and mask wearing among a large cohort of Chinese adults. Our study also found that the relevant knowledge, risk perception, and attitude were among the strongest predictors of the four NPIs. Wearing a mask, among the four NPIs, was the predominating protective measure against a COVID-19 infection, with an added preventive effect among those who practiced all or part of the other three NPIs. Our findings of many different predictors on different personal NPIs may also provide the possibility for further tailored health policy interventions. The study also emphasizes the importance, at an international level, of sharing information in a collaborative way to learn from everyone’s experiences about what interventions worked well and what were the impact of issues that may have resulted in poor outcomes such as delayed and misinformed actions.

## Appendix

Multimedia Appendix 1 COVID-19 questionnaire: Chinese version.

Multimedia Appendix 2 COVID-19 questionnaire: English version.

Multimedia Appendix 3 eTables.

## Abbreviations

AUROC: area under the receiver operating characteristics curve
NPI: nonpharmaceutical intervention
OR: odds ratio
RR: risk ratio
