# Supplementary material for: Relationship Between COVID-19 Infection and Risk Perception, Knowledge, Attitude, and Four Nonpharmaceutical Interventions During the Late Period of the COVID-19 Epidemic in China: Online Cross-Sectional Survey of 8158 Adults
Source: J Med Internet Res. 2020 Nov 13;22(11):e21372. doi: 10.2196/21372 (PMC7669364; doi:10.2196/21372)
Supplement: Multimedia Appendix 1 [file jmir_v22i11e21372_app1.docx]

# 居民对新冠疫灾的认识和健康相关行为的调查

各位朋友：

您好！为了解新冠肺炎疫情中广大居民对疫情认知、健康行为等情况，维护居民身心健康，促进国家防疫体制进步。期待您积极参与本次问卷调查，共同抗疫。请您根据自身真实感受填写答案。

本调查采取匿名填写方式，确保您所填信息保密。感谢您的支持！

华中科技大学同济医学院

重庆医科大学

新冠疫情调研项目组

2020年2月22日

**一、基本信息**

**个人信息：**

1.您的性别？

A.男

B.女

2.您的年龄 岁。

3.您的身高 cm。

4.您的现居地是 （下拉选择）

5.您居住在农村/城镇?

A.农村

B.城镇

6.您的最高学历是？

A.小学及以下

B.初中

C.高中

D.专科

E.本科

F.硕士及以上

7.您的职业是？

A.国家公务员

B.专业技术人员

C.职员

D.企业管理人员

E.工人

F.农民

G.学生

H.现役军人

I.自由职业者

J.个体经营者

K.退（离）休人员

L.医生

M.教师

N.无业人员

8.您是否与伴侣共同居住？

1. 是
2. 否

C.没有伴侣

9.您的家庭目前共同居住 人。（包括自己）

10.您的家庭平均每月收入为： （7位数）元。

11.您是家庭中的？

A.男主人

B.女主人

C.未成年孩子

D.成年青年（比如大学生、待业青年）

E.老年人

G.其他

12.您的家庭成员中是否有医务工作者？

A.是

B.否（跳至第15题）

13.该医务工作者是否参与了一线抗疫工作？

A.是

B.否

14.该医务工作者在本次抗疫中的岗位是？

A.诊治医生

B.护士

D.辅助科室人员

E.管理人员

F.其他

15.您的家庭成员中是否有参与本次联防联控工作？

A.有

B.没有（跳至17题）

16.该位联防联控人员是？

A.一线医护人员（直接接触病人或疑似病人）

B.二线医护人员（不直接接病人或疑似病人）

C.政府/事业单位人员

D.社区工作者

E.交通管制人员

F.警务人员

G.后勤物资供应人员

H.其他

**二、新冠核心防护知识**

17.您是否知道以下新冠肺炎核心防护措施？

17.11出门戴口罩

A.是

B.否 （跳答至17.21）

C.不清楚（跳答至17.21）

17.12您是否重复使用了口罩？

A.是

B.否

17.21勤洗手

1. 是
2. 否 （跳答至17.31）

C.不清楚（跳答至17.31）

17.22疫情发生后，您有增加洗手次数/时间吗？

A.有

B.无

17.31家庭室内勤通风

1. 是
2. 否 （跳答至17.41）
3. 不清楚（跳答至17.41）

17.32疫情发生后，您有做到家庭每天室内通风2小时以上吗？

A.是

B.否

17.41咳嗽/打喷嚏时，应用纸巾盖住口鼻，然后扔掉纸巾并洗手

A.是

B.否 （跳答至17.5）

C.不清楚（跳答至17.5）

17.42您咳嗽/打喷嚏时是否采取以上措施？

A.是

B.否

17.5避免与任何发烧和咳嗽的人密切接触

A.是

B.否

C.不清楚

17.6外出时与他人间隔1米以上距离

A.是

B.否

C.不清楚

17.7避免食用生鲜或未煮熟的动物产品

A.是

B.否

C.不清楚

18.疫情发生前，您是否具备传染病防护知识？

A. 完全不了解

B. 了解一点

C. 比较了解

D. 非常了解

**三、健康状况及健康相关行为**

19.您是否被确诊患有新冠肺炎？

A是

B.否

20.您所居住小区（村）有无新冠肺炎确诊病例？

A.有

B.没有（跳至22题）

C.不清楚（跳至22题）

21.您所在的楼栋有无新冠肺炎确诊病例？

A.有

B.没有

C.不清楚

22.您有无近距离（1米以内）接触过新冠肺炎确诊或疑似病例？

A.有

B.无

C.不清楚

23.疫情期间，您是否注意保持膳食营养均衡？

1. 是
2. 否

C. 不清楚

24.疫情期间，您饮食的量属如下哪种情况：

1. 大吃大喝，比平时多很多
2. 比平时多一点
3. 几乎没有变化（跳答25题）
4. 比平时少一点（跳答24.2题）
5. 比平时少很多（跳答24.2题）

24.1您吃得更多一些是希望增强抵抗力来应对疫情吗？ A是 B不是

24.2您吃得更少一些是因为疫情而食欲不佳吗？ A是 B不是

25.疫情发生前，您的体重 斤；现在，您的体重 斤。(非必答题）

26.您在处理生食和熟食时，菜板刀具是否分开？

1. 是
2. 否
3. 不清楚

27.您在处理生食和熟食之间是否洗手？

1. 是
2. 否
3. 不清楚

28.疫情对您的饮食行为有影响吗？

A有很大影响，饮食更健康了 B有一点好的影响 C基本无影响 D有一点不好的影响，E有很大的不好影响

29.疫情期间，您最近一周的身体活动情况是？

A.很少活动

B.中等强度的活动（如家务、跳舞、快走等）超过2.5个小时

C 高强度的活动（如跑步、游泳、篮球等）超过75分钟

D 中等强度和高强度活动混搭，超过2.5小时

30.疫情对您的身体活动行为有影响吗？

A有很大影响，更重视了 B有一点好的影响 C基本无影响 D有一点不好的影响，E有很大不好的影响

31.您是否吸烟？（吸烟指过去一个月里吸烟，一生中吸烟数量超过100支）

A.是

B.否（跳答至33题）

32.疫情期间您吸烟有变化吗？

A比平时多很多 B比平时多一点 C没变化 D比平时少一点 E比平时少很多

33.您是否饮酒？（饮酒指最近一个月饮过酒）

A.现在饮酒

B.现已戒酒（跳答至35题）

C.不喝酒（跳答至35题）

34.疫情期间您饮酒有变化吗？

A比平时多很多 B比平时多一点 C没变化 D比平时少一点 E比平时少很多

35.下面一些问题是关于您最近1个月的睡眠情况，请选择填写最符合您近1个月实际情况的答案。请回答下列问题：

（1） 近1个月，晚上上床睡觉通常（ ）点钟。

　　（2）近1个月，从上床到入睡通常需要（ ）分钟。

　　（3）近1个月，通常早上（ ）点起床

（4）近1个月，每夜通常实际睡眠（ ）小时(不等于卧床时间)。

对下列问题请选择1个最适合您的答案。

　　（5）近1个月 ，因下列情况影响睡眠而烦恼：

　　a. 入睡困难(30分钟内不能入睡)(1)无 (2)〈1次/周 (3)1-2次/周 (4)≥ 3次/周

　　b. 夜间易醒或早醒 (1)无 (2)〈1次/周 (3)1-2次/周 (4)≥ 3次/周

　　c. 夜间去厕所 (1)无 (2)〈1次/周 (3)1-2次/周 (4)≥ 3次/周

　　d. 呼吸不畅 (1)无 (2)〈1次/周 (3)1-2次/周 (4)≥ 3次/周

　　e. 咳嗽或鼾声高 (1)无 (2)〈1次/周 (3)1-2次/周 (4)≥3次/周

　　f. 感觉冷 (1)无 (2)〈1次/周 (3)1-2次/周 (4)≥3次/周

　　g. 感觉热 (1)无 (2)〈1次/周 (3)1-2次/周 (4)≥3次/周

　　h. 做恶梦 (1)无 (2)〈1次/周 (3)1-2次/周 (4)≥ 3次/周

　　i. 疼痛不适 (1)无 (2)〈1次/周 (3)1-2次/周 (4)≥ 3次/周

j. 其它影响睡眠的事情 (1)无 (2)〈1次/周 (3)1-2次/周 (4)≥ 3次/周

　　（6）近1个月，总的来说，您认为自己的睡眠质量(1)很好 (2)较好 (3)较差 (4)很差

　　（7）近1个月，您用药物催眠的情况(1)无 (2)〈1次/周 (3)1-2次/周 (4)≥ 3次/周

　　（8）近1个月，您常感到困倦吗 (1)无 (2)〈1次/周 (3)1-2次/周 (4)≥ 3次/周

（9）近1个月，您做事情的精力不足吗(1)没有 (2)偶尔有 (3)有时有 (4)经常有

36.您的睡眠受疫情影响吗？

A有很大影响 B有一点影响 C没有影响

出门行为：

1. 年三十和初一这两天，家里和亲戚朋友有聚餐吗？

A有 B没有 C不清楚

38.年三十那天后，您家里谁的**宅家**时间最长？

A.男主人

B.女主人

C.未成年孩子

D.成年青年（比如大学生、待业青年）

E.老年人

F.均未出门

38.1其最长宅家时间为______天。

39.其最长时间在家里的主要原因是？

A.主动/被动隔离

B.对疫情的恐惧

C.家里重点保护对象

D.没有口罩

E.身体健康欠佳

F.响应政府号召

G.其他

40.年三十那天后，您的家人中谁最经常出门？

A.男主人

B.女主人

C.未成年孩子

D.成年青年（比如大学生、待业青年）

E.老年人

F.均未出门（跳答至44题）

41.该成员最常出门的原因是？

1. 购买生活物资
2. 参与抗疫工作
3. 参与本职工作

D.取快递

E.聚会/聚餐

F.出门散步

G.其他

42.您外出时采取了以下哪些防护措施？

1. 佩戴口罩
2. 避免用手直接触摸眼、口、鼻
3. 避免与门把手等物品直接接触或事先消毒
4. 刻意与人保持距离，避免直接接触
5. 远离或背对咳嗽者
6. 以上均无
7. 一直没有出门

43.疫情期间，您外出时的想法是？

1. 害怕感染，快去快回
2. 和平时一样
3. 出门不易，多待一会
4. 一直没有出门

44.您宅家期间，业余活动有？（可多选）

A.看手机

B.看书

C.打游戏/麻将等娱乐活动

D.看电视

E.其他

45.疫情期间，您是否有学习（包括学业/职教/党教/自主学习等广泛的学习）？

A. 是

B. 否

46.本次疫情对您的学习产生了什么样的影响？

A.不适应学习方式与学习环境改变

B.学习热情不足，效果不好
C.不具有学习条件
D.无影响
E.拥有更多时间学习，学习状态更好

47.疫情期间，您有过焦虑、恐惧等情况吗？

A有过，很严重 B有过，一点儿 C没有

48.疫情期间，您得到的关心和安慰的来源有：

A无任何来源

B下列来源（可选多项）

A、配偶；B、其他家人；C、亲戚；D、朋友/同学D；E、同事；F、工作单位；G、党团工会等官方或半官方组织；H、宗教、社会团体等非官方组织；I、社区

49.目前您的心理健康状态是

A. 健康

B. 不健康

C. 不清楚

50.您如何评价自己在本次疫情中的心理适应能力？

A. 很不适应

B. 有一点不适应

C. 一般

D适应好

E适应得非常好

**四、疫灾中信息关注**

51.疫情期间，您对以下内容的关注程度是？

1. 病情病例变化情况
2. 非常关注 B.比较关注 C.一般 D.不怎么关注 E.没有关注

（2）疾病救治、药物研发及医护信息

A.非常关注 B.比较关注 C.一般 D.不怎么关注 E.没有关注

（3）疫情科普知识/权威专家解读

A.非常关注 B.比较关注 C.一般 D.不怎么关注 E.没有关注

（4）政府的政策及措施

A.非常关注 B.比较关注 C.一般 D.不怎么关注 E.没有关注

1. 捐款/捐物情况

A.非常关注 B.比较关注 C.一般 D.不怎么关注 E.没有关注

52.随着时间的推移，您对新冠疫情的关注度有无变化？

A.随时间增加

B.随时间减少

C.一直非常关注

D.一直轻度关注

E.从不关注

53.您最近一周，关注疫情相关信息的时间占休息时间的比例？

A.0~20%

B.21%~40%

C.41%~60%

D.61%~80%

E.81%~100%

54.关于本次疫情，您想了解的信息都能查到吗？

A.完全查不到

B.小部分能

C.一半能

D.大部分能

E.几乎都能

55.您通常通过哪些途径获取本次疫情相关信息？（多选）

A.官方网站、APP资讯及电视等公众传媒

B.个人的微博或微信朋友圈

C.QQ/微信群

D.亲朋好友

E.其他_______

56.关于本次疫情，您认为以下哪种信息来源途径最权威？

A.官方网站、APP资讯及电视等公众传媒

B.个人的微博或微信朋友圈

C.微信群或QQ群

D.亲朋好友

E.其他_______

57.关于本次疫情，您对以下哪种途径来源的信息最感兴趣？

A.官方网站、APP资讯及电视等公众传媒

B.个人的微博或微信朋友圈

C.微信群或QQ群

D.亲朋好友

E.其他_______

**五、对疫灾的感知与备灾、受灾情况**

58.面对新冠肺炎疫情，目前我们国家采取的突发公共卫生事件响应等级是？

1. Ⅰ级
2. Ⅱ级（跳答至61题）
3. Ⅲ级（跳答至61）
4. Ⅳ级（跳答至61）
5. 不清楚（跳答至61）

59.您认为Ⅰ级响应等级针对哪类事件？

1. 特别重大事件
2. 重大事件
3. 较大事件
4. 一般事件
5. 不清楚

60.您认为是否有必要启动Ⅰ级响应？

A. 是

B. 否

C. 不清楚

61.在您所在地，您认为以下哪些防疫措施有实施必要？（多选）

1. 封城
2. 出门者强制佩戴口罩
3. 暂停公共交通运营
4. 县/乡/村封路
5. 封闭小区
6. 延长假期
7. 强制隔离疑似患者
8. 所有疫区返乡人员隔离14天
9. 暂停所有娱乐场所/景区营业
10. 均无必要
11. 均有必要

62.哪一次事件使您意识到了本次疫情的严重性？

A.武汉卫健委发布新冠肺炎第一例死亡病例

B.钟南山院士表明新冠病毒存在“人传人”现象

C.武汉市政府宣布“封城”

D.所在地政府实施Ⅰ级响应

E.所在小区/村实施严格管控

F.其他

G.个人认为疫情不严重

63.您在平时是否有备灾意识？

A. 是

B. 否

64.您或您的家庭平时是否有储备应急物资？

A. 是

B. 否（跳至66题）

65.应急物资主要是为哪种灾害准备的？（多选）

A. 火灾

B. 地震

C. 洪灾

D. 疫病

E. 其他

66.您或您的家庭是否预留应急资金？

A. 是

B. 否

C. 不清楚

67.这次疫情发生以来，您是否在第一时间储备了物资？

A. 是

B. 否（跳答至69）

68.您在第一时间储备的物资是（指已买到的）？（多选）

1. 口罩、消毒液等防护用品
2. 食品
3. 非食品类生活物资
4. 药物
5. 其他

69.您认为您家里的备灾物资是否充分？

1. 非常不充分
2. 不充分
3. 一般
4. 充分
5. 非常充分

70.您目前最紧缺的物资是？

1. 口罩
2. 医用酒精/消毒液
3. 食品
4. 药物
5. 非食品类生活物资
6. 体温计
7. 其他
8. 没有紧缺物资

71.您或您的家庭是否因本次疫情受到了严重影响？

1. 是
2. 否（跳至73题）

72.最严重的影响是？

A.经济收入

B.家庭成员健康

C.学业/工作进度

D.他人歧视

E.其他

**六、对抗疫工作的态度及满意度评价**

73.疫情防控期间，您是否赞同“疫情防控，人人有责，人人尽责”？

A.赞同

B.不赞同

C.不清楚

74.您是否积极配合疫情防控工作？

A. 是

B. 否

75.您对所在地实施抗灾响应的反应速度是否满意？

A.十分不满意

B.不满意

C.一般

D.满意

E.十分满意

F.不清楚

76.您对现居住地公共交通管控力度是否满意？

A.十分不满意

B.不满意

C.一般

D.满意

E.十分满意

F.不清楚

77.您认为目前我国采取的防控力度是否合适？

1. 刚好合适
2. 力度过大，应减小力度
3. 力度过小，应加大力度
4. 不清楚

78.您对国家现阶段疫情防控工作总体是否满意？

A.十分不满意

B.不满意

C.一般

D.满意

E.十分满意

F.不清楚

79.面对这次疫情，您最想说的话是：_________________________
