# Supplementary material for: Relationship Between COVID-19 Infection and Risk Perception, Knowledge, Attitude, and Four Nonpharmaceutical Interventions During the Late Period of the COVID-19 Epidemic in China: Online Cross-Sectional Survey of 8158 Adults
Source: J Med Internet Res. 2020 Nov 13;22(11):e21372. doi: 10.2196/21372 (PMC7669364; doi:10.2196/21372)
Supplement: Multimedia Appendix 2 [file jmir_v22i11e21372_app2.docx]

# A survey of residents' awareness and health-related behaviors of the COVID-19 epidemic

Dear all,

Hello everyone! In order to understand the cognition and health behaviors, maintain the physical and mental health of the residents and improve the national epidemic prevention system during the Coronavirus disease 2019 (COVID-19) epidemic, we look forward to your active participation in this questionnaire survey to fight the epidemic together. Please fill in the answers faithfully based on your true feelings.

This survey is anonymous to ensure the confidentiality of your information. Thank you for your participation and support!

Tongji Medical College, Huazhong University of Science and Technology

Chongqing Medical University

February 22, 2020

### Basic information

1. Your gender?

A. Male B. Female

2.Your age is  years old.

3. Your height is cm.

4. Your current residential area is in_______ (dropdown option) (such as Wanzhou, Chongqing)

5. Are you living in a countryside or town?

A. Countryside

B. Town

6.What is your highest academic qualification ？

A. Primary school and below

B. Junior school

C. Middle school

D. Professional college

E. Undergraduate

F. Postgraduate or above

7. What is your occupation?

A. National civil servants

B. Professional and Technical Personnel

C. Company employee

D. Enterprise Managers

E. Worker

F. Farmer

G. Student

H. Active Military Personnel

I. Freelancer

J. Self-employed

K. Retired personnel

L. Doctor

M. Teacher

N. Unemployed

8.Do you live with your partner?

A. Yes

B. No

C. No partner

9.How many people in your family currently live together (including yourself)?

10.What is the average monthly income of your family? yuan.

11. What is your role in the family?

A. Male host

B. Hostess

C. Underage children

D. Adult youth (such as college students, unemployed youth)

E. Elderly

G. Other

12.Are there any health professional workers in your family?

A. Yes

B. No

13. Is the health professional worker involved in the frontline anti-epidemic work?

A. Yes

B. No

14. What is the position of the health professional worker in this fight against the epidemic?

A. Doctor

B. Nurse

D. Auxiliary department staff

E. Management

F. Other

15.Have any of your family members participated in the Joint Prevention and Joint Control community work？

A. Yes

B. No（skip to 17）

16.What is the job of this Joint Prevention and Joint Control worker?

A. Front-line health workers (Direct contact with patients or suspected patients)

B. Second-line health staff (No direct contact with patients or suspected patients)

C. Government or public institution staff

D. Community workers

E. Traffic Control Personnel

F. Police personnel

G. Logistic material supply staff

H. Others

### COVID-19 core protection knowledge

17. Are you familiar with the following protective measures?

17.11 Do you wear a face mask when you go out?

A. Yes

B. No (skip to 17.21)

C. Not sure (skip to 17.21)

17.12 Do you reuse the mask？

A. Yes

B. No

17.21 Do you wash hands frequently？

A. Yes

B. No (skip to 17.31)

C. Not sure (skip to 17.31)

17.22 After the COVID-19 outbreak, have you increased the number/times of hand washing?

A. Yes

B. No

17.31 Do you know that indoor ventilation should be more than 2 hours per day？

A. Yes

B. No (skip to 17.41)

C. Not sure (skip to 17.41)

17.32 After the COVID-19 outbreak, have you done indoor ventilation more than 2 hours per day？

A. Yes

B. No

17.41 Do you know that when coughing/sneezing, cover your mouth and nose with a tissue, then throw away the tissue and wash your hands?

A. Yes

B. No (skip to 17.5)

C. Not clear (skip to 17.5)

17.42 Do you take the above measures when you coughed/ sneezed?

A. Yes

B. No

C. Never cough/ sneeze during this period

17.5 Do you know to avoid close contact with anyone who has fever and cough?

A. Yes

B. No

C. Not sure

17.6 Do you know that 1 meter above social distance is needed when you go out?

A. Yes

B. No

C. Not sure

17.7 Avoid eating raw or undercooked animal products?

A. Yes

B. No

C. Not sure

18. Before the COVID-19 outbreak, did you have knowledge of infectious disease prevention?

A, Not at all

B. A little

C. Quite well

S. Very well

1. **Health status and health-related behaviors**

19.Have you been diagnosed with COVID-19?

A. Yes

B. No

20.Are there any confirmed cases of COVID-19 in your community/ village?

A. Yes

B. No

C. Not sure

21.Are there any confirmed cases of COVID-19 in your building?

A. Yes

B. No

C. Not sure

22.Have you been in close contact (within 1 meter) of confirmed or suspected cases of COVID-19？

A. Yes

B. No

C. Not sure

23. During the epidemic, do you pay attention to maintaining a balanced diet?

A. Yes

B. No

C. Not sure

24. During the epidemic, what is the amount of your food?

A. much more than usual

B. A little more than usual

C. Almost no change (skip to answer 25 questions)

D. A little less than usual (skip to answer 24.2 questions)

E. Much less than usual (skip to answer 24.2 questions)

24.1 Do you eat more because you want to strengthen your resistance to deal with the COVID-19?

A. Yes

B. No

24.2 Are you eating less because of the poor appetite caused by the epidemic situation?

A. Yes

B. No

25.Before the COVID-19 outbreak, you weight was____; now, you weight is___. (optional question)

26. Do you use chopping board/knifes separately when preparing raw and cooked food?

A. Yes

B. No

C. Not sure

27. Do you wash your hands between preparing raw and cooked food?

A. Yes

B. No

C. Not sure

28. Does the COVID-19 affect your eating behavior?

A. Has a great influence, and - healthier diet.

B. Has a little good influence

C. Has no influence

D. Has a little bad influence

E. Has a lot bad influence.

29. During the COVID-19 outbreak period, what was your physical activity level in the last week?

A. Very little activity

B. Medium-intensity activities (such as housework, dancing, brisk walking, etc.) more than 2.5 hours

C. High-intensity activities (such as running, swimming, basketball, etc.) exceed 75 minutes

D. Mixed medium intensity and high intensity activities, more than 2.5 hours

30. Does the epidemic affect your physical activity behavior?

A Has a great positive influence

B. Has a little good influence

C. Has no influence

D. Has a little bad influence

E. Has a lot bad influence.

31.Do you smoke? (Smoking refers to smoking in the past month, more than 100 cigarettes in a lifetime)

A. Yes

B. No (skip to 33)

32.Have you changed your smoking habit during the COVID-19 outbreak?

A. Smoking much more than usual

B. Smoking a little more than usual

C. No change

D. Smoking a little less than usual

E. Smoking much less than usual

33.Do you drink alcohol? (Drinking refers to drinking alcohol in the last month)

A. Yes

B. Have stopped drinking now (skip to 35)

C. Don't drink (skip to 35)

34. Have you changed your drinking habit during the COVID-19 outbreak?

A. Drinking more than usual

B. Drinking a little more than usual

C. No change

D. Drinking a little less than usual

1. Drinking much less than usual

35.The following questions are about your sleep in the past month. Please choose the answer that best fits your actual situation in the past month. please answer the following question:

(1) In the past month, I usually went to bed at _____o'clock at night.

(2) In the past month, I usually took _____ minutes from going to bed to falling asleep.

(3) In the past month, I usually got up at _____ in the morning.

(4) In the past month, I usually slept for _____ hours per night (not equal to bedtime).

For the following questions, please choose the one that suits you best.

(5) In the past 1 month, I had trouble with sleeping because of the following conditions.

a. Difficulty falling asleep (not falling asleep within 30 minutes)

(1) None (2) <1 time/week (3) 1-2 times/week (4) ≥ 3 times/week

b. nighttime awakening or early awakening

(1) None (2) <1 time/week (3) 1-2 times/week (4) ≥ 3 times/week

c. Going to the toilet at night

(1) None (2) <1 time/week (3) 1-2 times/week (4) ≥ 3 times/week

d. Breathlessness

(1) None (2) <1 time/week (3) 1-2 times/week (4) ≥ 3 times/week

e. noisy of cough or snoring

(1) None (2) <1 time/week (3) 1-2 times/week (4) ≥3 times/week

f. Feeling cold

(1) None (2) <1 time/week (3) 1-2 times/week (4) ≥3 times/week

g. Feeling hot

(1) None (2) <1 time/week (3) 1-2 times/week (4) ≥3 times/week

h. Nightmares

(1) None (2) <1 time/week (3) 1-2 times/week (4) ≥ 3 times/week

i. Pain and discomfort

(1) None (2) <1 time/week (3) 1-2 times/week (4) ≥ 3 times/week

j. Other things that affect sleep

(1) None (2) <1 time/week (3) 1-2 times/week (4) ≥ 3 times/week

(6) In the past month, generally speaking, you think your sleep quality is

(1) very good (2) good (3) poor (4) poor

(7) In the past month, how often have you taken sleeping pills

(1) None (2) <1 time/week (3) 1-2 times/week (4) ≥ 3 times/week

(8) In the past month, have you often felt sleepy

(1) None (2) <1 time/week (3) 1-2 times/week (4) ≥ 3 times/week

(9) In the past month, did you have insufficient energy to do things?

(1) No (2) Occasionally (3) Sometimes (4) Often

36. Is your sleep affected by the COVID-19 epidemic?

A. a great influence

B. a little influence

C. no influence

**Outdoor behaviors:**

37. Were there any dinners with relatives and friends at home on the New Year’s Eve and the first day of the new year?

A. Yes

B. No

C. Not sure

38.After the Chinese New Year eve (i.e. 24 Jan 2020), who in your family has the longest stay-at-home time?

A. Male host

B. Hostess

C. Minor children

D. Adult youth (e.g. college students, unemployed youth)

E. Elderly

F. All did not go out

38.1. The maximum length of stay at home is ______ days.

39. What is the main reason for the longest stay at home?

A. Active/passive isolation

B. Fear of the epidemic

C. Key protection targets

D. No mask

E. Poor health

F. In response to the Government's call

G. Other

40.After New Year’s Eve, who goes out most often in your family？

A. Host

B. Hostess

C. Minor children

D. Adult youth（such as, college students）

E. Elders

F. No one goes out（skip to 44）

41.Why does this member go out most often?

A. Purchase living supplies

B. Participation in the fight against the epidemic

C. Work

D. Take express delivery

E. Gathering

F. Take a walk

G. Other

42.When you went out, which of the following protective measures do you take? (Multiple option)

1. Wear a face mask
2. Avoid touching eyes, mouth and nose directly with your hands
3. Avoid direct contact with door handles and other items or disinfect beforehand
4. Deliberately keep a distance from others and avoid direct contact
5. Stay away from or turn your back to the coughing person
6. None of the above
7. Never go out

43. During the epidemic, what was your thought when you went out?

A. Fear of infection, get back immediately

B. Same as usual

C. It's not easy to go out, stay out longer

D. Never goes out

44. During your stay at home, what are your spare time activities? (Multiple choice)

A. Playing with mobile phone

B. Reading books

C. Playing games/Mahjong and other entertainment activities

D. Watching TV

E. Other

45. During the COVID-19 outbreak, do you study (including extensive studies such as academic/vocational education/independent study)?

A. Yes

B. No

46. What is the impact of this epidemic on your study?

A. Not adapting to changes in learning methods and learning environment

B. Lack of enthusiasm for learning and poor results

C. Poor learning conditions

D. No effect

E. Have more time to learn, and learn better

47.During the COVID-19 outbreak, do you feel anxiety or fear?

A. Yes

B. No

48. During the COVID-19 outbreak, the sources of care and comfort you received are:

A. Has no source

B. The following sources (multiple options can be selected)

a. Spouse; b. Other family members; c. Relatives; d. Friends/classmates; e. Colleagues; f. Workplaces; g. Official or semi-official organizations such as party, youth league and trade union; h. Non-official organizations such as religious and social organizations; i. Community

49. What is your current mental health status?

A. Health

B. Unhealthy

C. Not clear

50. How do you think your psychological adaptability during this epidemic?

A. Very poor

B. A little bit poor C. Fair

D Adapted well

E Adapted very well

### Information source during the epidemic

51. During the epidemic, how concerned were you on the following content?

(1) Changes in disease cases

A. Very concerned

B. Quite concerned

C. Fairly concerned

D. Not very concerned

E. Not concerned

(2) Disease treatment, drug development and medical information

A. Very concerned

B. Quite concerned

C. Fairly concerned

D. Not very concerned

E. Not concerned

(3) COVID-19 epidemic popular science knowledge/authoritative expert interpretation

A. Very concerned

B. Quite concerned

C. Fairly concerned

D. Not very concerned

E. Not concerned

(4) Government policies and measures

A. Very concerned

B. Quite concerned

C. Fairly concerned

D. Not very concerned

E. Not concerned

(5) Situation of donations/materials

A. Very concerned

B. Quite concerned

C. Fairly concerned

D. Not very concerned

E. Not concerned

52. Over time, has your attention to the COVID-19 changed?

A. Increased over time

B. Decreased over time

C. Has always been very concerned

D. Has always been slightly concerned

E. Never paid attention

53. In the past week, what percentage of your time did you spend paying attention to information about COVID-19 in your leisure time?

A. 0~20%

B.21%~40%

C.41%~60%

D. 61%~80%

E.81%~100%

54. Regarding COVID-19, can you find all the information you want to know?

A. None of the information

B. A small amount of the information

C. Half of the information

D. Most of the information

E. Almost all

55. How do you usually obtain information about COVID-19? (Multiple choice)

A. Official website, APP information, TV and other public media

B. Personal Weibo or WeChat Moments

C.QQ/WeChat group

D. Family and friends

E. Other_______

56. Regarding COVID-19, which of the following sources of information do you think is the most authoritative?

A. Official website, APP information, TV and other public media

B. Personal Weibo or WeChat Moments

C. WeChat group or QQ group

D. Family and friends

E. Other_______

57. Regarding COVID-19, which of the following sources of information are you most interested in?

A. Official website, APP information, TV and other public media

B. Personal Weibo or WeChat Moments

C. WeChat group or QQ group

D. Family and friends

E. Other_______

### Perception and preparedness of the epidemic

58. In the face of the COVID-19, what is the current level of public health emergencies response adopted by our country?

A. level I

B. level Ⅱ (skip to 61 questions)

C. level Ⅲ (skip to 61)

D. Level IV (Skip to 61)

E. Not sure (skip to 61)

59. What kind of incident do you think the Level I response is for?

A. Especially important events

B. Major events

C. Larger events

D. General events

E. Not sure

60. Do you think it is necessary to initiate Level I response?

A. Yes

B. No

C. Not sure

61. In your location, which of the following epidemic prevention measures do you think are necessary? (Multiple choice)

A. Lockdown the city

B. People who go out are obliged to wear a mask

C. Suspend public transportation operations

D. County/township/village road closure

E. Lockdown community

F. Extend holidays

G. Compulsory isolation of suspected patients

H. All returnees from affected areas are quarantined for 14 days

I. Suspend all entertainment venues / scenic spots

J. None necessary

K. All necessary

62. Which incident made you aware of the seriousness of COVID-19?

A. Wuhan Health Commission reported the first COVID-19 related pneumonia death case

B. Dr. Zhong Nanshan stated that the COVID-19 has “human-to-human transmission"

C. Wuhan Municipal Government announced the "closure of the city"

D. Local government implements Level I response

E. Strict control in the community/village

F. Other

G. Personally think the epidemic is not serious

63. Do you have disaster preparedness awareness in normal time?

A. Yes

B. No

64. Do you or your family usually stockpile emergency supplies?

A. Yes

B. No (skip to question 66)

65. What kind of disasters are emergency stockpile prepared for? (Multiple choice)

A. Fire

B. Earthquake

C. Flood

D. Disease

E. Other

66. Do you or your family reserve emergency funds?

A. Yes

B. No

C. Not sure

67. Since the outbreak of this epidemic, have you stockpiled supplies at the very beginning?

A. Yes

B. No (skip to 69)

68. What suppliers did you reserve at the very beginning (referring to the purchased ones)?

(Multiple choice)

A. Protective equipment such as masks and disinfectants

B. Food

C. Non-food daily living products

D. Drugs

E. Other

69.Do you think the disaster preparedness materials in your home are sufficient?

1. Very inadequate
2. Inadequate
3. Not quite adequate
4. Adequate
5. Very adequate

70.What supplies are you in most shortage of?

1. Face mask
2. Medical alcohol/ disinfectant
3. Food
4. Medicine
5. Non-food daily living products
6. Thermometer
7. Other
8. No shortage

71.Have you or your family been seriously affected by the COVID-19 epidemic?

1. Yes
2. No（skip to 73）

72.What is the most serious affect？

A. Financial income

B. Health of family members

C. Study/ work progress

D. Prejudice by others

E. Other

### Attitude towards the epidemic

73.During the epidemic period, do you agree that “It is everyone’s responsibility to prevent and control the epidemic.”?

A. Agree

B. Disagree

C. Not sure

74.Do you actively cooperate with the epidemic prevention and control work?

A. Yes

B. No

75. Are you satisfied with the speed of the local disaster response?

A. Very dissatisfied

B. Not satisfied

C. Not quite satisfied

D. Satisfied

E. Very satisfied

F. Not sure

76. Are you satisfied with the degree of public transportation control in your current residential area?

A. Very dissatisfied

B. Not satisfied

C. Not quite satisfied

D. Satisfied

E. Very satisfied

F. Not sure

77. Do you think the current prevention and control measures adopted by our country are appropriate?

A. Just right

B. Too strong, should be reduced

C. Too weak, should be increased

D. Not sure

78. Are you in general satisfied with the country's current epidemic prevention and control work?

A. Very dissatisfied

B. Not satisfied

C. Not quite satisfied

D. Satisfied

E. Very satisfied

F. Not sure

79. With respect to this COVID-19 epidemic, what you want to say most is________
