# Supplementary material for: Relationship Between COVID-19 Infection and Risk Perception, Knowledge, Attitude, and Four Nonpharmaceutical Interventions During the Late Period of the COVID-19 Epidemic in China: Online Cross-Sectional Survey of 8158 Adults
Source: J Med Internet Res. 2020 Nov 13;22(11):e21372. doi: 10.2196/21372 (PMC7669364; doi:10.2196/21372)
Supplement: Multimedia Appendix 3 [file jmir_v22i11e21372_app3.docx]

**Appendix 2:**

**eTable 1a. ORs and their 95%CI from logistic regression models for four NPIs (Continued).**

|  | **Handwashing (yes/no)** | | **Proper coughing (yes/no)** | |
| --- | --- | --- | --- | --- |
| Predictors | **OR** | **95%CI** | **OR** | **95%CI** |
| Age groups |  |  |  |  |
| 18-39 years | - | - | - | - |
| 40-59 years | 1.46 | (0.84 - 2.53) | 2.24*** | (1.56 - 3.23) |
| >=60 years | 0.83 | (0.27 - 2.51) | 2.50 | (0.97 - 6.44) |
| Sex (female vs male) | 1.12 | (0.82 - 1.53) | 0.99 | (0.78 - 1.27) |
| Living in city (vs rural) | 1.67*** | (1.26 - 2.22) | 0.97 | (0.76 - 1.22) |
| Education |  |  |  |  |
| Primary school | - | - | - | - |
| High school | 2.22 | (0.75 - 6.53) | 2.72* | (1.20 - 6.16) |
| Professional college | 3.12 | (0.97 - 10.09) | 5.77*** | (2.39 - 13.91) |
| University/post-graduate | 1.68 | (0.55 - 5.15) | 2.92* | (1.25 - 6.83) |
| Occupation |  |  |  |  |
| Health professionals | - | - | - | - |
| Government payee | 0.41 | (0.15 - 1.15) | 0.78 | (0.44 - 1.38) |
| Factory workers/managers | 0.23** | (0.08 - 0.66) | 0.59 | (0.33 - 1.08) |
| Farmers | 0.31 | (0.08 - 1.19) | 0.80 | (0.32 - 2.00) |
| Students | 0.10*** | (0.03 - 0.28) | 0.30*** | (0.16 - 0.55) |
| Others | 0.28* | (0.09 - 0.86) | 0.71 | (0.37 - 1.37) |
| Family monthly Income |  |  |  |  |
| 0/1000 RMB | - | - | - | - |
| 1001/2000 RMB | 1.45 | (0.82 - 2.56) | 0.74 | (0.47 - 1.19) |
| 2001/4000 RMB | 1.13 | (0.70 - 1.81) | 0.72 | (0.47 - 1.09) |
| 4001/6000 RMB | 0.84 | (0.52 - 1.34) | 0.89 | (0.57 - 1.37) |
| 6001/8000 RMB | 1.51 | (0.77 - 2.96) | 0.91 | (0.53 - 1.56) |
| 8001/10000 RMB | 0.86 | (0.50 - 1.48) | 0.70 | (0.44 - 1.13) |
| 10001/20000 RMB | 0.82 | (0.43 - 1.57) | 0.61 | (0.36 - 1.02) |
| 20001/4000000 RMB | 1.02 | (0.39 - 2.70) | 0.60 | (0.30 - 1.20) |
| Not sure/unanswered | 1.24 | (0.59 - 2.59) | 0.57 | (0.32 - 1.00) |
| Smoked during the last month (no vs yes) | 0.86 | (0.48 - 1.54) | 0.96 | (0.64 - 1.45) |
| Drank alcohol during the last month |  |  |  |  |
| Yes | - | - | - | - |
| Gave up | 0.58 | (0.25 - 1.34) | 1.46 | (0.75 - 2.86) |
| Don't drink | 0.95 | (0.57 - 1.56) | 1.75** | (1.25 - 2.45) |
| Body Mass Index |  |  |  |  |
| Underweight | - | - | - | - |
| Normal | 1.23 | (0.89 - 1.71) | 0.91 | (0.70 - 1.18) |
| Overweight | 0.75 | (0.45 - 1.23) | 0.94 | (0.63 - 1.40) |
| Obese | 0.48 | (0.20 - 1.12) | 0.82 | (0.35 - 1.90) |
| Not available | 1.15 | (0.72 - 1.83) | 0.92 | (0.63 - 1.33) |
| Currently living with a partner? |  |  |  |  |
| Yes | - | - | - | - |
| No | 0.61 | (0.36 - 1.01) | 0.60** | (0.42 - 0.85) |
| I don't have a partner | 0.57* | (0.35 - 0.95) | 0.59** | (0.41 - 0.83) |
| Family member who is a health professional? (no vs yes) | 0.94 | (0.62 - 1.43) | 0.71* | (0.51 - 0.98) |
| Living in the area with community infection |  |  |  |  |
| Hubei Province | - | - | - | - |
| Outside Hubei Province with 100+ cases | 1.19 | (0.58 - 2.46) | 2.65*** | (1.65 - 4.25) |
| Other | 1.15 | (0.58 - 2.28) | 2.19*** | (1.41 - 3.39) |
| Family member who is part of the local community efforts against COVID-19 (no vs yes) | 0.62* | (0.41 - 0.94) | 0.58*** | (0.43 - 0.78) |
| When perceived that the situation was serious |  |  |  |  |
| After the lockdown of Wuhan City | - | - | - | - |
| Before the lockdown of Wuhan City | 1.43* | (1.08 - 1.90) | 1.54*** | (1.24 - 1.92) |
| Agree that the task of fighting against COVID-19 is everyone’s responsibility | 5.59*** | (2.64 - 11.82) | 1.75 | (0.78 - 3.93) |
| Know why and how to act properly (yes vs no) | 22.60*** | (9.31 - 54.83) |  |  |
| Constant | 0.78 | (0.11 - 5.64) | 3.57 | (0.88 - 14.51) |
| Observations | 8,036 |  | 6,349 |  |
| AUROC^§^ | 0.831 |  | 0.804 |  |

Note: *** *P*<0.001, ** *P*<0.01, * *P*<0.05

§: AUROC: The Area Under the Receiver Operating Characteristic Curve

**eTable 1b: ORs and their 95%CI from logistic regression models for four NPIs**

|  | **Social distancing(yes/no)** | | **Mask Wearing(yes/no)** | |
| --- | --- | --- | --- | --- |
| Predictors | **OR** | **95%CI** | **OR** | **95%CI** |
| Age groups |  |  |  |  |
| 18-39 years | - | - | - | - |
| 40-59 years | 0.76** | (0.64 - 0.91) | 1.08 | (0.65 - 1.82) |
| >=60 years | 0.51*** | (0.35 - 0.73) | 0.25** | (0.10 - 0.63) |
| Sex (female vs male) | 1.27** | (1.08 - 1.50) | 0.74 | (0.43 - 1.26) |
| Living in city (vs rural) | 0.93 | (0.79 - 1.10) | 1.86** | (1.18 - 2.94) |
| Education |  |  |  |  |
| Primary school | - | - | - | - |
| High school | 1.08 | (0.67 - 1.76) | 0.59 | (0.10 - 3.32) |
| Professional college | 1.30 | (0.79 - 2.16) | 0.46 | (0.08 - 2.73) |
| University/post-graduate | 1.31 | (0.79 - 2.19) | 0.68 | (0.11 - 4.16) |
| Occupation |  |  |  |  |
| Health professionals | - | - | - | - |
| Government payee | 0.85 | (0.67 - 1.08) | 1.32 | (0.72 - 2.43) |
| Factory workers/managers | 0.81 | (0.61 - 1.06) | 1.22 | (0.57 - 2.60) |
| Farmers | 0.89 | (0.59 - 1.33) | 0.75 | (0.24 - 2.38) |
| Students | 0.61** | (0.44 - 0.86) | 1.24 | (0.48 - 3.19) |
| Others | 1.00 | (0.73 - 1.35) | 1.24 | (0.50 - 3.11) |
| Family monthly Income |  |  |  |  |
| 0/1000 RMB | - | - | - | - |
| 1001/2000 RMB | 0.91 | (0.68 - 1.23) | 1.06 | (0.46 - 2.47) |
| 2001/4000 RMB | 1.01 | (0.77 - 1.33) | 1.03 | (0.48 - 2.22) |
| 4001/6000 RMB | 1.01 | (0.76 - 1.35) | 1.19 | (0.53 - 2.69) |
| 6001/8000 RMB | 1.14 | (0.81 - 1.60) | 2.12 | (0.71 - 6.33) |
| 8001/10000 RMB | 1.35 | (0.97 - 1.87) | 2.90 | (0.93 - 9.03) |
| 10001/20000 RMB | 1.11 | (0.77 - 1.58) | 0.98 | (0.36 - 2.66) |
| 20001/4000000 RMB | 1.14 | (0.71 - 1.85) | 1.52 | (0.36 - 6.50) |
| Not sure/unanswered | 1.19 | (0.79 - 1.81) | 0.96 | (0.25 - 3.70) |
| Smoked during the last month (no vs yes) | 1.39** | (1.14 - 1.71) | 1.71 | (0.94 - 3.11) |
| Drank alcohol during the last month |  |  |  |  |
| Yes | - | - | - | - |
| Gave up | 1.17 | (0.82 - 1.67) | 0.31** | (0.13 - 0.72) |
| Don't drink | 1.12 | (0.91 - 1.37) | 0.83 | (0.44 - 1.57) |
| Body Mass Index |  |  |  |  |
| Underweight | - | - | - | - |
| Normal | 1.21* | (1.01 - 1.46) | 1.19 | (0.72 - 1.95) |
| Overweight | 1.03 | (0.82 - 1.29) | 1.12 | (0.57 - 2.18) |
| Obese | 1.26 | (0.73 - 2.16) | 0.92 | (0.21 - 4.04) |
| Not available | 1.00 | (0.78 - 1.29) | 2.02 | (0.86 - 4.75) |
| Currently living with a partner? |  |  |  |  |
| Yes | - | - | - | - |
| No | 1.23* | (1.00 - 1.51) | 1.28 | (0.70 - 2.35) |
| I do not have a partner | 1.64*** | (1.29 - 2.08) | 0.82 | (0.43 - 1.55) |
| Family member who is a health professional? (no vs yes) | 0.89 | (0.74 - 1.08) | 1.57 | (0.91 - 2.69) |
| Living in the area with community infection |  |  |  |  |
| Hubei Province | - | - | - | - |
| Outside Hubei Province with 100+ cases | 0.72 | (0.48 - 1.08) | 1.08 | (0.38 - 3.08) |
| Other | 0.71 | (0.48 - 1.05) | 1.36 | (0.48 - 3.83) |
| Family member who is part of the local community efforts against COVID-19 (no vs yes) | 1.11 | (0.94 - 1.30) | 0.85 | (0.53 - 1.36) |
| When perceived that the situation was serious |  |  |  |  |
| After the lockdown of Wuhan City | - | - | - | - |
| Before the lockdown of Wuhan City | 0.91 | (0.80 - 1.05) | 1.00 | (0.67 - 1.50) |
| Agree that the task of fighting against COVID-19 is everyone’s responsibility | 3.76*** | (2.10 - 6.71) | 26.89*** | (11.66 - 62.03) |
| Know why and how to act properly (yes vs no) | 4.26*** | (3.03 - 5.97) |  |  |
| Constant | 0.33* | (0.13 - 0.87) | 0.96 | (0.10 - 9.51) |
| Observations | 8,036 |  | 5,054 |  |
| AUROC^§^ | 0.637 |  | 0.750 |  |
| Note: *** *P*<0.001, ** *P*<0.01, * *P*<0.05  §: AUROC: The Area Under the Receiver Operating Characteristic Curve | | | | |

**eTable 2: Self-isolation**

| Questions: | N=8158 | % |
| --- | --- | --- |
| Had a party during New Year’s Eve and New Year (2 days) |  |  |
| Yes | 1833 | 22.5% |
| No | 6242 | 76.5% |
| Not sure | 83 | 1.0% |
| The major reason for staying at home |  |  |
| Self/compulsory insolation | 1107 | 13.6% |
| Fear of the virus | 469 | 5.7% |
| Focused on family protection | 310 | 3.8% |
| No mask | 242 | 3.0% |
| Ill health | 63 | 0.8% |
| Complying with the call from government | 5354 | 65.6% |
| Other | 613 | 7.5% |
| The main reason for going out* |  |  |
| Shopping | 2073 | 40.5% |
| Involved in fighting COVID-19 | 1643 | 32.1% |
| Usual employment | 1098 | 21.4% |
| Receiving delivery | 38 | 0.7% |
| Socializing/dinner party | 17 | 0.3% |
| Going for a walk | 131 | 2.6% |
| Other | 120 | 2.3% |
| What did you do when you went out* |  |  |
| Shortened the time to avoid infection | 3798 | 74.2% |
| As usual | 1000 | 19.5% |
| Stayed longer given the hassle | 50 | 1.0% |
| Uncertain | 272 | 5.3% |

*Only including 5,120 respondents who went out after the outbreak of COVID-19.
